# Cropping system regulates the optimal organic substitution ratio via soil-plant nitrogen and phosphorus transformation and utilization

**DOI:** 10.3389/fpls.2026.1877524

**Published:** 2026-08-06

**Authors:** Yaqian Yan, Haiyang Jin, Yuan Huang, Hang Song, Jiarui Wang, Baoting Fang, Fei Zheng, Haifang Pang, Xiwen Yang, Xiangdong Li, Dexian He

**Affiliations:** 1 College of Agronomy, Henan Agricultural University, Zhengzhou, China; 2 Wheat Research Institute, Henan Academy of Agricultural Sciences, Zhengzhou, China

**Keywords:** cropping system, nutrient transformation, organic substitution, wheat, yield

## Abstract

**Introduction:**

The optimal of organic fertilizer for substituting chemical fertilizer in a specific planting system is unclear. This study aimed to clarify how organic substitution regulates nitrogen (N) and phosphorus (P) transformation and utilization in the soil–wheat system under different cropping systems.

**Methods:**

This study investigated three organic fertilizer substitution (0%, 25%, 50%) on N and P transformation and utilization in the soil-wheat environment under maize-wheat (MW), peanut-wheat (PW), and soybean-wheat (SW) cropping systems. Indicators related to rhizosphere soil N (N-acq) and P-acquiring (P-acq) enzyme activities, wheat root N and P metabolism enzyme activities, N and P nutrient accumulation and utilization, and yield.

**Results:**

Organic substitution reduced the N-acq enzyme activity in the rhizosphere soil (2.50%-35.32%) and the mineral N (N_min_) content, but increased the P-acq enzyme activity (0.81% to 18.15%) and the available P (AP) content (0.68%-31.51%). It also enhanced the N (NR, GS, GDH, GOGAT) and P metabolism (ACP) enzymes in wheat roots, thereby promoting the accumulation of N and P. Under the MW, the 25% substitution rate performed best root N and P metabolism enzyme activities, N and P-use efficiency (NUE, PUE), and grain N and P accumulation. Meanwhile, under the SW and PW, the 50% substitution rate was superior.

**Discussion:**

The key drivers of yield formation were N related parameters under the MW and P related parameters under the SW and PW. Present study, 25% organic substitution is suitable for the MW cropping system, while 50% suits SW and PW cropping systems.

## Graphical Abstract

**Figure:**
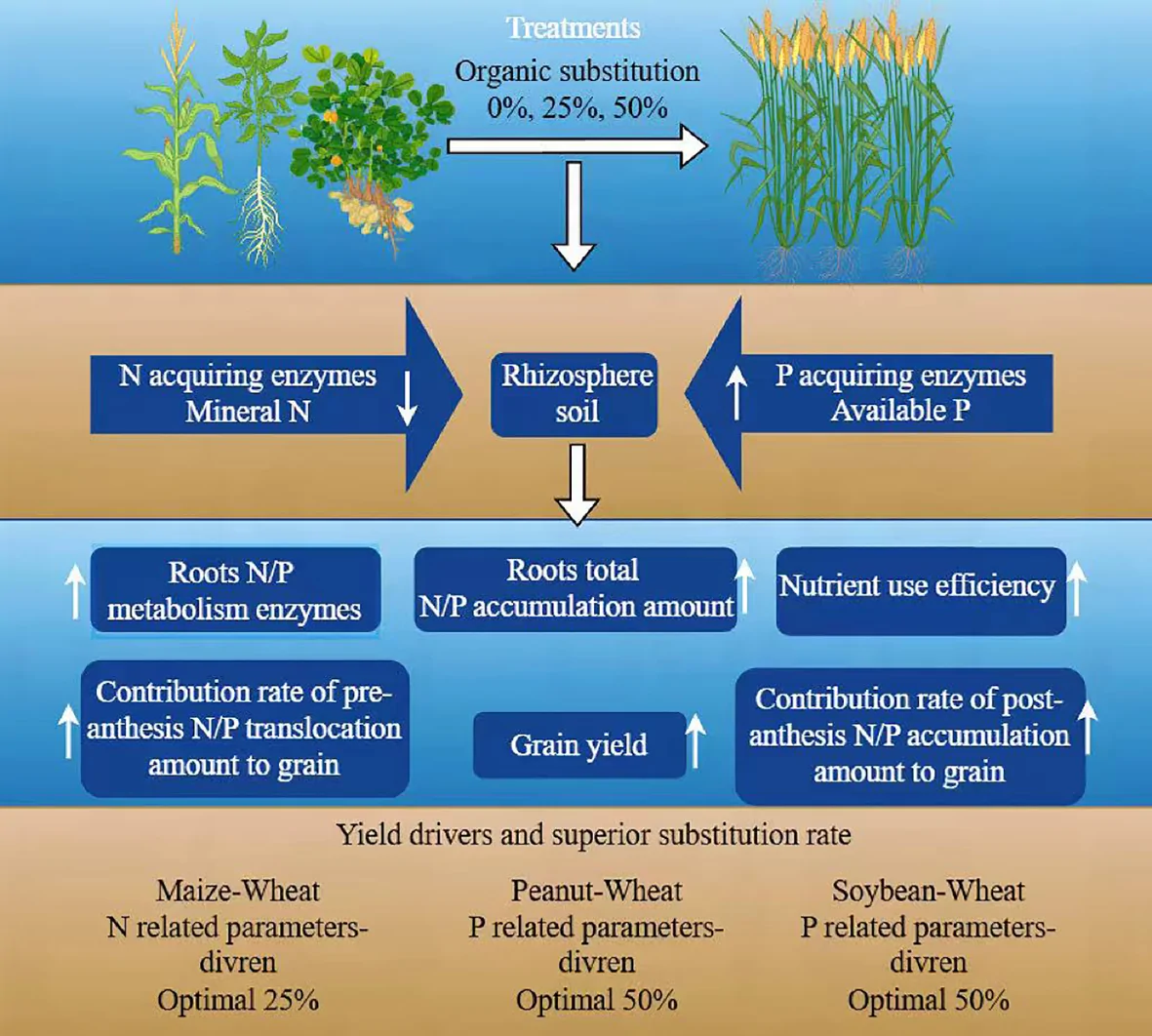

## 1 Introduction

Nitrogen (N) and phosphorus (P) are essential nutrients that play vital roles in regulating growth, development, and yield formation in plants, such as wheat (Ahmadi et al., 2024). Typically, the transformation, uptake, and accumulation of N and P within the soil-plant system determine crop productivity. However, long-term and excessive use of chemical fertilizers, especially N and P fertilizers, negatively affects soil quality and causes environmental problems (Fang et al., 2026; Wu et al., 2020). Excessive N is lost via leaching and volatilization, resulting in environmental issues such as nitrate pollution of groundwater, ammonia (NH_3_) volatilization from farmland, and nitrous oxide (N_2_O) emissions (Zhou et al., 2023). Meanwhile, the accumulation of P leads to a P surplus in the soil and elevates the risk of eutrophication (surface water bodies) (Tian et al., 2022). Therefore, how to reduce fertilizer application through scientific methods while improving fertilizer use efficiency and promoting stable, high crop yields has become a key strategy for achieving long-term agricultural sustainability.

Organic fertilizers are increasingly used as sustainable alternatives to chemical fertilizers. The organic fertilizer substitution alleviates the adverse effects of long-term chemical fertilizer use. The substitution specifically improves soil health (Huang et al., 2023), reduces greenhouse gas emissions (Li et al., 2021), and enhances crop yield and productivity (Wu et al., 2024b). Moreover, the abundant organic carbon in organic fertilizers facilitates the proliferation of soil microorganisms and enhances their activity. This effect, in turn, can augment the synthesis and secretion of soil N-acquiring and P-acquiring (N-acq and P-acq) enzymes, expedite the transformation of organic N and P into inorganic forms, and offer a continuous and stable nutrient supply for crop growth (Chang et al., 2024). Gao et al. (2023) found that the partial substitution of manure decreased N_min_ concentration and enhanced BG and LAP activities compared with chemical fertilizer alone.

Recent studies have shown that partial substitution of N fertilizers with organic fertilizers promotes nutrient absorption and transport to grains, improving crop yield and utilization (Liu et al., 2023a; Xie et al., 2017; Zhai et al., 2022). However, multiple factors, such as the organic fertilizer type, substitution ratio, soil type, crop species, and cropping systems, influence the efficacy of organic fertilizer replacing chemical fertilizer (Liu et al., 2021b). Thus, the optimal replacement ratio varies across conditions (Hijbeek et al., 2018). A high rate of organic fertilizer substitution results in inadequate nutrient availability due to their slow-release characteristics, potentially failing to meet crop growth requirements and leading to reduced yields (Huang et al., 2016). Meanwhile, a low substitution ratio may not fully leverage the advantages of organic fertilizers in improving soil quality and enhancing nutrient-utilization efficiency (Ren et al., 2023). Substituting 37.5% of the basal chemical fertilizer with organic fertilizer increased the agronomic efficiency of maize by 22.8% and improved N fertilizer partial productivity by 11.5% (Meng et al., 2025). Therefore, determining the optimal proportion of organic fertilizer for substituting chemical fertilizer in a specific planting system and studying the mechanism by which it regulates yield formation through N and P transformation and utilization are crucial for maximizing efficacy.

The North China Plain is a major region for grain production in China, and winter wheat- summer maize is the main intensive cropping systems. Due to long-term winter wheat- summer maize cropping system and chemical fertilizer application to maintain high yields, soil quality is deteriorating (Yan et al., 2024). This seriously threatens production stability. Over the years, legume cultivation, crop rotation, and organic amendments have been widely adopted as sustainable practices to mitigate the environmental impacts of agricultural activities (Poulton et al., 2018; Yan et al., 2025). Studies have demonstrated that incorporating leguminous crops into cereal-based cropping systems increases soil available P content, enhances crop N and P uptake, and improves grain yields (Ghosh et al., 2020; Yu et al., 2021). Compared with non-leguminous crops, leguminous crops in a planting system enhance the yield of subsequent crops (Zhang et al., 2025). A deeper understanding of how different legume-wheat rotation patterns affect wheat yield will provide scientific support for sustainably enhancing this cropping system.

Studies so far have primarily focused on the effects of organic fertilizer substitution within single cropping systems or on the use of individual types of organic fertilizers. However, the interaction between the cropping system and organic fertilizer substitution and its influence on rhizosphere soil N and P transformation, plant N and P uptake and utilization, and subsequent yield formation in wheat remains unclear. Therefore, the present study carried out a two-year-long field experiment in an open-field setting to (1) characterize rhizosphere N and P transformation, wheat nutrient uptake and yield responses to organic substitution ratios across cropping systems, and screen the optimal substitution rate for each system. (2) identify the key factors affecting wheat yield under different planting systems and organic fertilizer substitution rates; and (3) reveal how cropping system interacts with organic substitution to regulate rhizosphere N and P transformation, facilitate nutrient translocation, and raise nutrient use efficiency and maintain grain yield.

## 2 Materials and methods

### 2.1 Site description

In this study, field experiments were conducted at the Suiping Institute of Agricultural Sciences in Zhumadian City, Henan Province (China; 35°35′ N, 114°13′E) from October 2021 to June 2023. Monthly mean temperature and total precipitation throughout the experimental years were visualized in **Supplementary Figure 1**. The area has a Vertisol soil. Before the experiment, the basic properties of the 0–20 cm soil layer were as follows: 8.34 g·kg^-^¹ organic carbon, 1.03 g·kg^-^¹ total N, 0.30 g·kg^-^¹ total P, 15.28 g·kg^-^¹ total potassium (K), and 4.60 pH.

### 2.2 Experimental design

This experiment was set up using three fertilizer treatments, namely 0% organic fertilizer substitution (F1), 25% of chemical fertilizer substituted with organic fertilizer (F2), and 50% of chemical fertilizer substituted with organic fertilizer (F3), under the maize-wheat (MW), soybean-wheat (SW), and peanut-wheat (PW) rotation cropping systems. Thus, the experiment had 9 treatments, each replicated 3 times, resulting in 27 plots, measuring 48 m^-2^ each. Urea was used as the N fertilizer in this study, single superphosphate as the P fertilizer, and potassium chloride as the K fertilizer. Meanwhile, cow manure was used as the organic fertilizer. The nutrient composition of the organic fertilizer used was as follows: 372.63 g·kg^-^¹ organic carbon, 2.36% N, 1.82% P_2_O_5_, 0.93% K_2_O and a C/N ratio of 41.53. The application rates of N, P, and K fertilizers across the treatments were calculated based on the nutrient content of the organic fertilizer and the substitution rate. The application rate of organic fertilizer is based on the dry weight equivalent. The fertilization doses for each crop are shown in **Table 1**. Wheat was sown in late October. Before sowing, basal fertilizer was applied uniformly to the soil surface and incorporated to a depth of 20 cm through rotary tillage. All field management practices, except the application rates of organic and nitrogen fertilizer, were consistent across the three treatments.

**Table 1:** Information on the fertilizer application rates in the experiment.

Crop	Fertilizers application	N kg·hm^-2^	P_2_O_5_ kg·hm^-2^	K_2_O kg·hm^-2^	Organic fertilizer kg·hm^-2^
Maize	100% chemical fertilizer	225.00	112.50	112.50	0.00
Peanut	100% chemical fertilizer	112.50	112.50	112.50	0.00
Soybean	100% chemical fertilizer	33.75	33.75	33.75	0.00
Wheat	100% chemical fertilizer	225.00	120.00	90.00	0.00
	25% organic substitution rate	168.71	76.63	67.86	2383.50
	50% organic substitution rate	112.50	33.25	45.63	4767.50

The nutrient application rates for wheat season in all plots were N 225 kg·hm^-2^, P_2_O_5–_120 kg·hm^-2^, and K 90 kg/ha.

### 2.3 Soil sample collection and analysis

Soil samples were collected during the jointing, anthesis, and filling stages of wheat from 2021 to 2023. After removing the topsoil, the whole wheat roots were collected from individual rows within each 33 cm sampling section, with all root samples taken from the 0–20 cm soil layer. Gently shaken to remove any excess surface soil of root systems, and then gently remove the 1–2 mm thick soil adhering to the root surface (Barillot et al., 2013). Each soil sample was further divided into three subsamples: labor one was maintained on ice and stored at −20 °C for subsequent determination of mineral nitrogen (N_min_); one was stored at 4 °C to assess the soil nutrient-acquiring enzyme activity; and one was air-dried, ground, and sieved to measure the soil available phosphorus (AP). Nitrate-N and ammonium-N were extracted from soil with 1 mol·L^-1^ KCl and analyzed with a flow analyzer (AMS Alliance, France), and N_min_ was calculated. The activities of the nutrient-acquiring enzymes, namely N-acetyl-β-D-glucosaminidase (NAG), leucine aminopeptidase (LAP), and phosphatase (Phos), were assessed using the fluorescent microplate assays (Lu et al., 2024). The activity of enzymes with similar functions was normalized. N-acq: mean of leucine aminopeptidase and N-acetyl-β-D-glucosaminidase (Luo et al., 2018).

### 2.4 Wheat sample collection and analysis

During the jointing, anthesis, and filling stages, wheat plants within a 33 cm area of each plot were selected. The roots within the top 0–20 cm soil layer were dug out, and all loose soil clumps were gently shaken off. Then, the activities of root acid phosphatase (ACP), glutamate dehydrogenase (GDH), glutamate synthase (GOGAT), glutamine synthetase (GS), and nitrate reductase (NR) were analyzed using the corresponding biochemical kits from Beijing Solarbio Science & Technology Co., Ltd. (Beijing, China).

The wheat root and straw were dried at high temperature and digested with concentrated sulfuric acid and hydrogen peroxide. Total N in the digested sample was measured using the Kjeldahl method, and total P was determined using the molybdenum antimony-scandium colorimetric method (Lu, 2000). During harvest, a 10 m^2^ wheat grain sample was collected from each plot to measure wheat yield. Additionally, the resource-use efficiencies of winter wheat, including N-use efficiency and P-use efficiency, were calculated using the following formulas (Li et al., 2023; Si et al., 2020; Zhang et al., 2024).

N uptake efficiency (NUE, kg·kg^-1^) = aboveground N accumulation at maturity/N application rate (1-1)

N partial factor productivity (NPFP, kg·kg^-1^) = grain yield/N application rate(1-2)

N harvest index (NHI, %) = grain N accumulation at maturity/aboveground N accumulation at maturity (1-3)

Pre-anthesis N translocation amount (kg·hm^-2^) = N accumulation of vegetative organs at anthesis − N accumulation of vegetative organs at maturity (1-4)

Pre-anthesis N transport rate (%) = Pre-anthesis N translocation amount/N accumulation of vegetative organs at anthesis (1-5)

Contribution rate of pre-anthesis N translocation amount to grain (%) = Pre-anthesis N translocation amount/N accumulation in grain at maturity (1-6)

Post-anthesis N accumulation amount (kg·hm^-2^) = aboveground N accumulation at maturity − aboveground N accumulation at anthesis (1-7)

Contribution rate of post-anthesis N accumulation amount to grain (%) = Post-anthesis N accumulation amount/N accumulation in grain at maturity(1-8)

Relevant calculation formulas for P are the same as above.

### 2.5 Statistical analysis

Different software packages were selected according to specific analytical purposes in this study. Data processing was performed using Excel 2010. Statistical analysis was conducted using SPSS software version 26.0 (IBM Co., Armonk, NY, USA), and Treatment differences were separated using the full-named Duncan’s Multiple Range Test (DMRT) at the significance level of *p* < 0.05. < The graphs were plotted with Origin 2024 (OriginLab, Northampton, MA). Using a Pearson correlation coefficient, the association among parameters was analyzed. Using a Random Forest (RF) model analysis (Svetnik et al., 2003) the key factors associated with the effects of organic substitution on wheat yield across different planting systems. The RF procedure was applied using the “RandomForest” package in R (version 3.3.3). Structural equation modeling (SEM) was used to reveal the direct effects and indirect pathways by which different cropping system and organic fertilizer substitution influence wheat nutrient uptake, translocation, and utilization efficiency—thereby driving grain yield formation—through their regulation of soil nutrient supply and nutrient transformation processes. The SEM analysis was performed by Amos 22 (AMOS IBM, USA), and the model’s fit was goodness when the output parameters reached the following standards. The goodness-of-fit for SEM was assessed by the chi-square (χ2) test and *p*-value, goodness-of-fit index (GFI), and root mean square error of approximation (RMSEA).

## 3 Results

### 3.1 Yield and yield components of wheat

In this study, both F2 and F3 treatments increased the wheat grain yield compared to F1 under the MW cropping system during the two-year period, while only F3 increased the wheat yield under the SW and the PW cropping systems (**Table 2**). Notably, F3 resulted in 0.86%–4.51% lesser yield than F2 under the MW cropping system. Under this system, F2 increased wheat yield primarily by increasing the 1000-grain weight. Meanwhile, under SW and PW cropping systems, F3 resulted in 1.73%–5.42% and 1.00%–2.29% increase in yield compared to F1, and 8.97%–15.02% and 1.79%–3.35% increase compared to F2. Here, F3 increased wheat yield mainly by increasing spike number and 1000-grain weight. The grain yield under F2 was significantly lower than that under F1 in these two systems.

**Table 2:** Grain yield and its components of wheat plants under different treatments.

Year	Treatments	Grain yield (kg·hm-2)	Spike number (×104·hm-2)	Grains per spike	1000-grain weight (g)
2021-2022	MWF1	9194.26 ± 31.64b	610.61 ± 34.11a	42.71 ± 2.75b	42.85 ± 0.54c
	MWF2	9691.84 ± 91.93a	544.95 ± 30.08b	45.74 ± 3.62a	44.18 ± 0.99b
	MWF3	9254.29 ± 118.08b	531.82 ± 34.11b	41.59 ± 2.57ab	47.70 ± 0.20a
	SWF1	9791.90 ± 144.70b	553.03 ± 31.49c	44.74 ± 1.40a	47.62 ± 1.30ab
	SWF2	9473.40 ± 90.63c	705.55 ± 25.46a	37.88 ± 1.15b	45.07 ± 1.78b
	SWF3	10322.82 ± 46.73a	598.23 ± 20.12b	43.83 ± 1.48c	49.83 ± 1.42a
	PWF1	9514.43 ± 81.54ab	581.82 ± 36.36b	45.09 ± 3.16a	41.82 ± 1.77b
	PWF2	9440.05 ± 256.73b	589.09 ± 27.74b	38.49 ± 174b	45.28 ± 0.40a
	PWF3	9609.47 ± 92.14a	656.32 ± 26.61a	35.33 ± 1.82c	45.35 ± 1.06a
2022-2023	MWF1	9613.14 ± 152.83a	543.33 ± 41.76b	43.45 ± 2.30a	44.81 ± 1.13b
	MWF2	9738.20 ± 28.88a	722.22 ± 30.09a	38.41 ± 2.28b	44.99 ± 1.17b
	MWF3	9654.83 ± 175.09a	755.05 ± 41.00a	35.29 ± 2.41b	48.68 ± 2.23a
	SWF1	10105.05 ± 90.18a	722.22 ± 69.17ab	44.53 ± 1.73a	38.50 ± 0.94b
	SWF2	8937.80 ± 76.41b	644.70 ± 30.18b	33.67 ± 1.56b	45.62 ± 1.89a
	SWF3	10280.14 ± 164.02a	702.78 ± 26.61a	35.32 ± 1.59b	46.76 ± 1.84a
	PWF1	9621.48 ± 38.21ab	671.72 ± 48.70ab	38.98 ± 3.20b	42.44 ± 0.45b
	PWF2	9521.43 ± 194.28b	601.47 ± 38.21b	41.83 ± 2.62a	38.75 ± 0.49c
	PWF3	9839.92 ± 128.23a	708.58 ± 20.12a	36.39 ± 3.06b	44.76 ± 1.55a
Treatment		*	*	*	*
Year		*	*	*	*
Treatment*Year		*	*	*	*

Means in a column for the same cropping system followed by different lowercase letters are significantly different (*p* < 0.05). *Mean significant difference at the 0.05 level.

### 3.2 Nutrient-acquiring enzyme activities and nutrient content of rhizosphere soil

**Figure 1:**
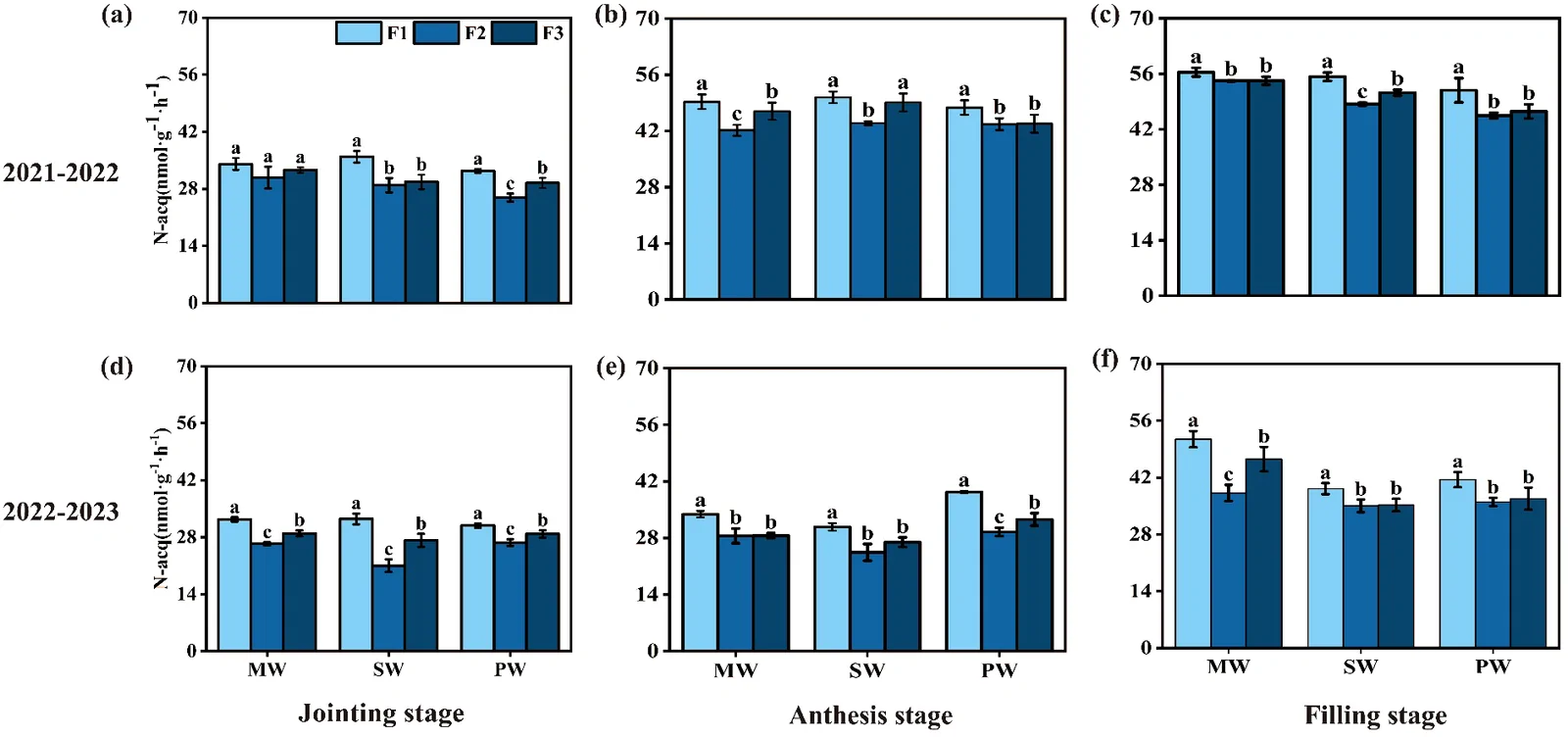
The activities of the N-acq enzymes in the rhizosphere soil of wheat at different growth stages. MW: maize-wheat cropping system; SW: soybean-wheat cropping system; PW: peanut-wheat cropping system; F1: 0% organic substitution; F2: 25% chemical fertilizer substituted with organic fertilizer; F3: 50% chemical fertilizer substituted with organic fertilizer. N-acq: mean of leucine aminopeptidase and *N*-acetyl-*β*-D-glucosaminidase. The **(a–c)** means results at jointing, anthesis and filling stages in the 2021–2022 growing season, while **(d–f)** correspond to the three growth stages in the 2022–2023 growing season. Different lowercase letters indicate significant differences between treatments under the same cropping (*p* < 0.05).

The N-acq enzymes in rhizosphere soil exhibited a progressive increase over the two years as the wheat growth stage advanced. Notably, the organic substitution treatments (F2 and F3) had lower N-acq enzyme activity than the chemical fertilizer treatment (F1) (**Figure 1**). Under all three cropping systems, the activity of N-acq enzymes in F2 and F3 decreased by 3.87%–35.32% and 2.50%–17.22%, respectively, compared with F1. Among the two organic fertilizer substitution treatments, F2 showed the greatest drop-in activity.

**Figure 2:**
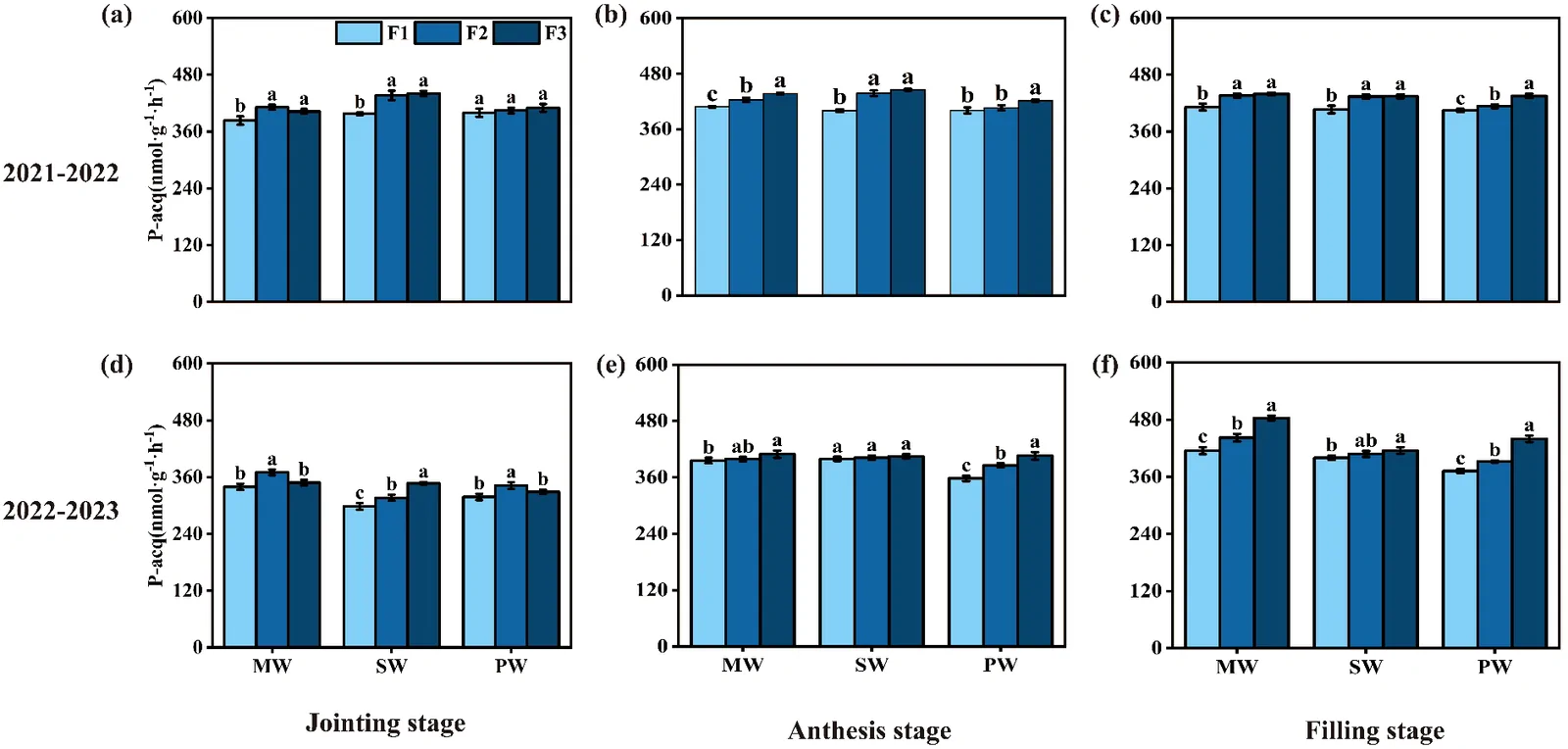
The activity of P-acq enzyme in the rhizosphere soil of wheat at different growth stages. MW: maize-wheat cropping system; SW: soybean-wheat cropping system; PW: peanut-wheat cropping system; F1: 0% organic substitution; F2: 25% chemical fertilizer substituted with organic fertilizer; F3: 50% chemical fertilizer substituted with organic fertilizer. The **(a–c)** means results at jointing, anthesis and filling stages in the 2021–2022 growing season, while panels **(d–f)** correspond to the three growth stages in the 2022–2023 growing season. Different lowercase letters indicate significant differences between treatments under the same cropping system (*p* < 0.05).

The P-acq enzyme activity in rhizosphere soil increased progressively over the two years as the wheat growth advanced. The organic substitution treatments increased P-acq enzyme activity compared with chemical fertilizer alone (F1) (**Figure 2**). During the two years, the activity of the P-acq enzyme under the three cropping systems increased in both F2 and F3 (0.81%–9.02% and 1.38%–18.15%, respectively). Specifically, during wheat anthesis and filling, the increase in enzyme activity was the greatest in F3.

**Figure 3:**
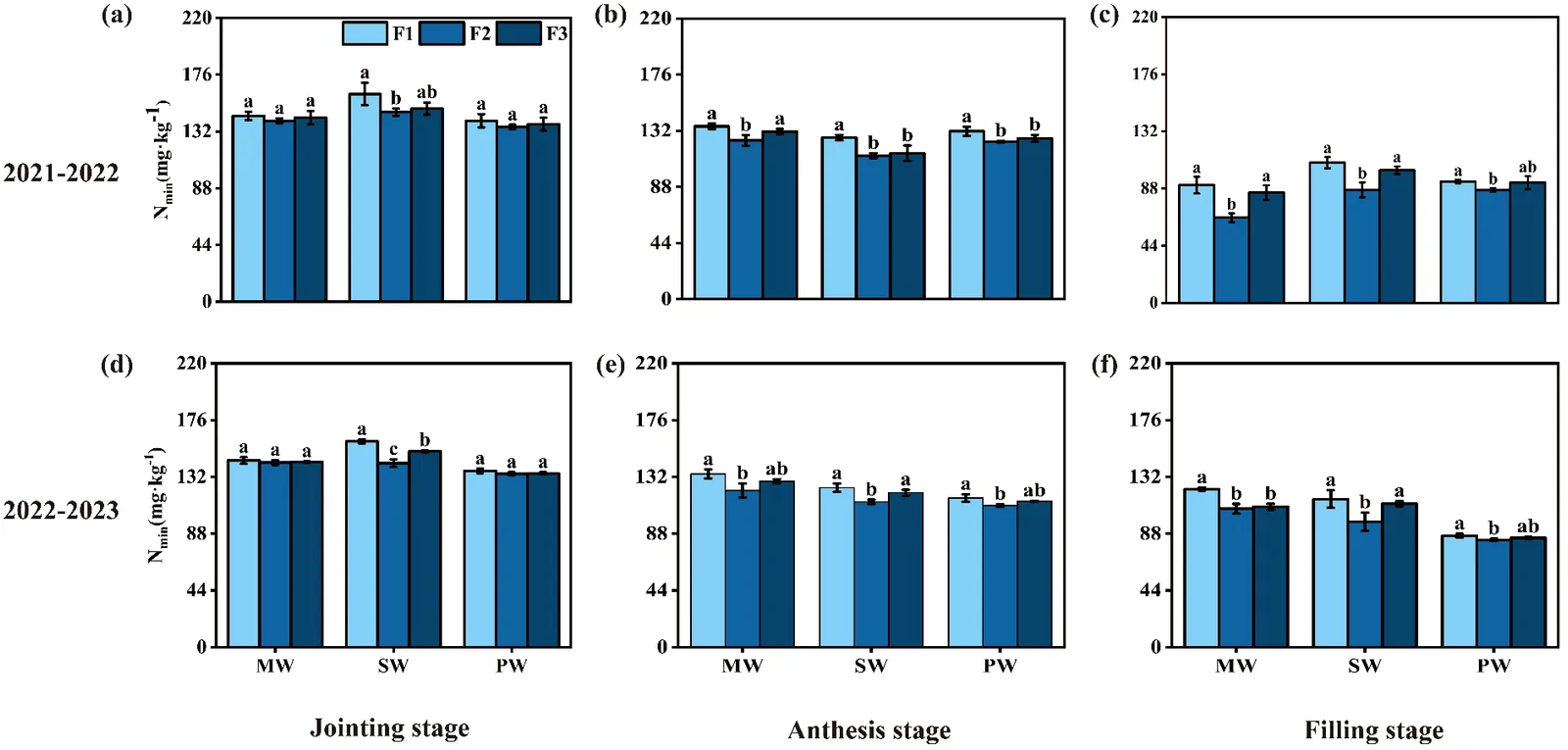
The N_min_ content of the rhizosphere soil of wheat at different growth stages. MW: maize-wheat cropping system; SW: soybean-wheat cropping system; PW: peanut-wheat cropping system; F1: 0% organic substitution; F2: 25% chemical fertilizer substituted with organic fertilizer; F3: 50% chemical fertilizer substituted with organic fertilizer. The **(a–c)** means results at jointing, anthesis and filling stages in the 2021–2022 growing season, while **(d–f)** correspond to the three growth stages in the 2022–2023 growing season. Different lowercase letters indicate significant differences between treatments under the same cropping system (*p* < 0.05).

**Figure 4:**
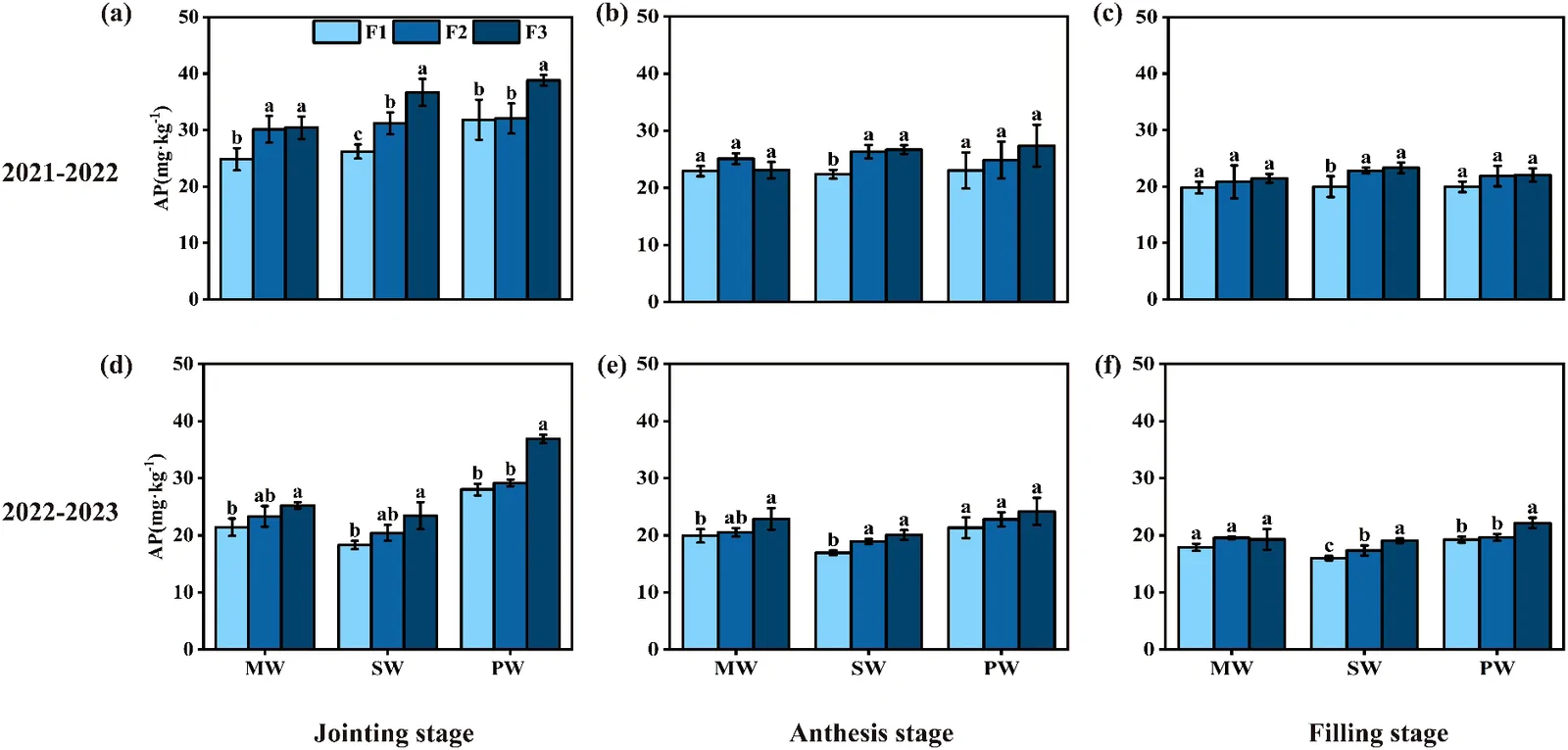
The AP content of the rhizosphere soil of wheat at different growth stages. MW: maize-wheat cropping system; SW: soybean-wheat cropping system; PW: peanut-wheat cropping system; F1: 0% organic substitution of chemical fertilizer; F2: 25% organic substitution of chemical fertilizer; F3: 50% organic substitution of chemical. The **(a–c)** means results at jointing, anthesis and filling stages in the 2021–2022 growing season, while **(d–f)** correspond to the three growth stages in the 2022–2023 growing season. Different lowercase letters indicate significant differences between treatments under the same cropping system (*p* < 0.05).

The content of N_min_ and AP in the rhizosphere soil exhibited a progressive decrease over the two-year period as the wheat growth advanced (**Figures 3**, **4**). During the period, N_min_ under the three cropping systems were low in both F2 and F3, which demonstrated a decrease of 3.87%–35.32% and 2.50%–17.22%, respectively, compared with F1 (**Figure 3**). Meanwhile, rhizosphere soil AP content under the three cropping systems was high in both F2 and F3 treatments, with 0.69%–21.22% and 0.68%–31.51% increase, respectively, compared with F1 (**Figure 4**). Overall, F3 resulted in the highest N_min_ and AP content across all three cropping systems.

### 3.3 The activities of enzymes associated with nutrient metabolism in the wheat roots

**Figure 5:**
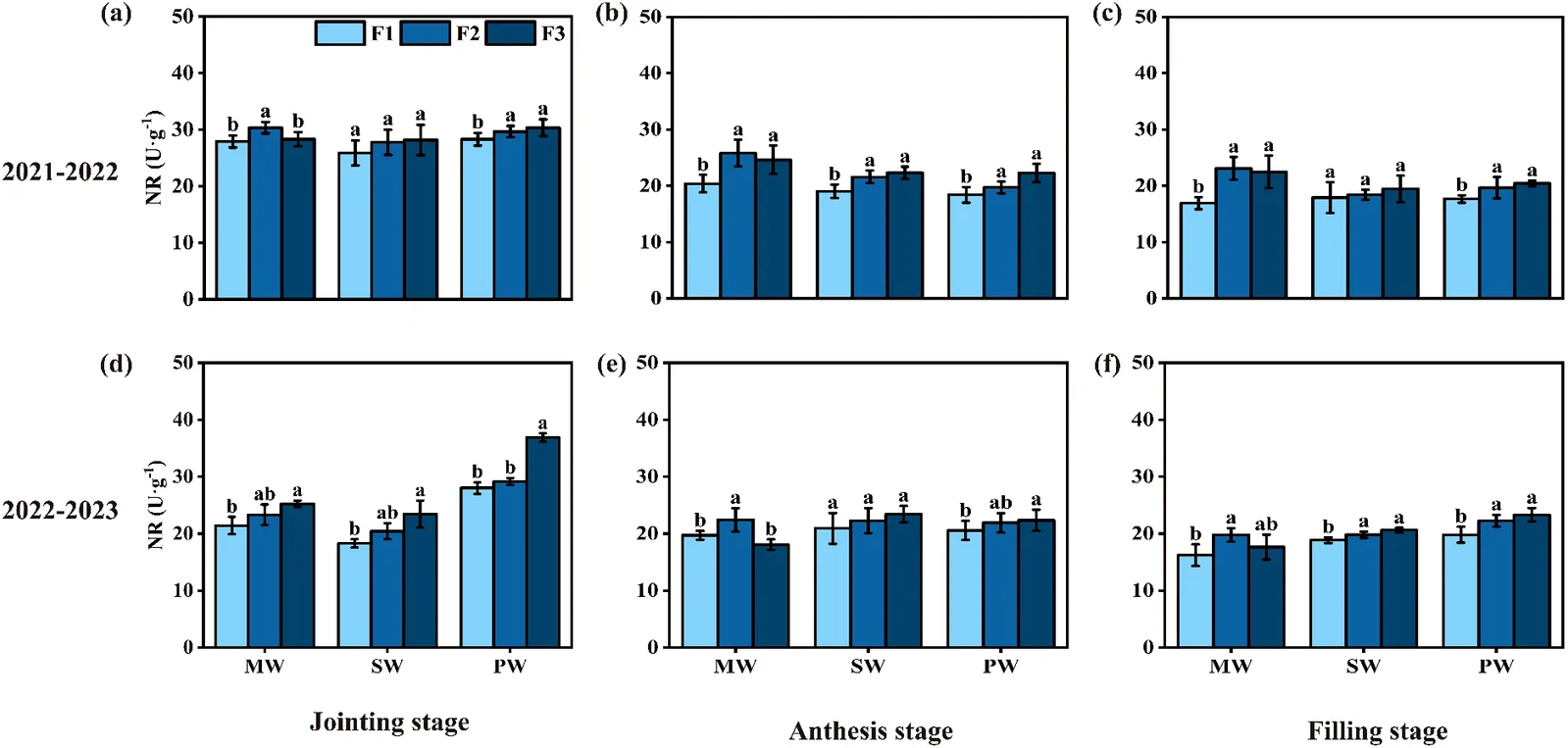
The nitrate reductase (NR) activity in wheat roots at different growth stages. MW: maize-wheat cropping system; SW: soybean-wheat cropping system; PW: peanut-wheat cropping system; F1: 0% organic substitution; F2: 25% chemical fertilizer substituted with organic fertilizer; F3: 50% chemical fertilizer substituted with organic fertilizer. NR: nitrate reductase. The **(a–c)** means results at jointing, anthesis and filling stages in the 2021–2022 growing season, while **(d–f)** correspond to the three growth stages in the 2022–2023 growing season. Different lowercase letters indicate significant differences between treatments under the same cropping system (*p* < 0.05).

The activities of enzymes associated with N metabolism in wheat roots increased with organic substitution treatments compared with F1 (**Figure 5**, **Supplementary Figures 2**–**4**). Under the MW cropping system, the activities of NR, GOGAT, GDH, and GS were significantly higher in F2 than in F1. Compared with F2, F3 decreased the activity of NR, GOGAT, GDH, and GS by 2.70%–19.47%, 0.56%–4.36%, 0.40%–6.23%, and 2.08%–5.29%, respectively. Compared with F1, F3 increased the activities of NR, GOGAT, GDH, and GS by 1.65%–10.94%, 3.77%–16.38%, 2.87%–11.54%, and 3.42%–15.15%, respectively. Meanwhile, under the SW cropping system, the higher organic substitution rates led to increased activities of NR and GDH. However, only F2 resulted in a significant increase in GOGAT and GS activities. Under the PW cropping system, F3 significantly increased the activities of NR, GOGAT, GDH, and GS compared with F1 (3.65%–21.34%, 6.95%–14.41%, 3.44%–17.03%, and 16.02%–56.94%, respectively).

**Figure 6:**
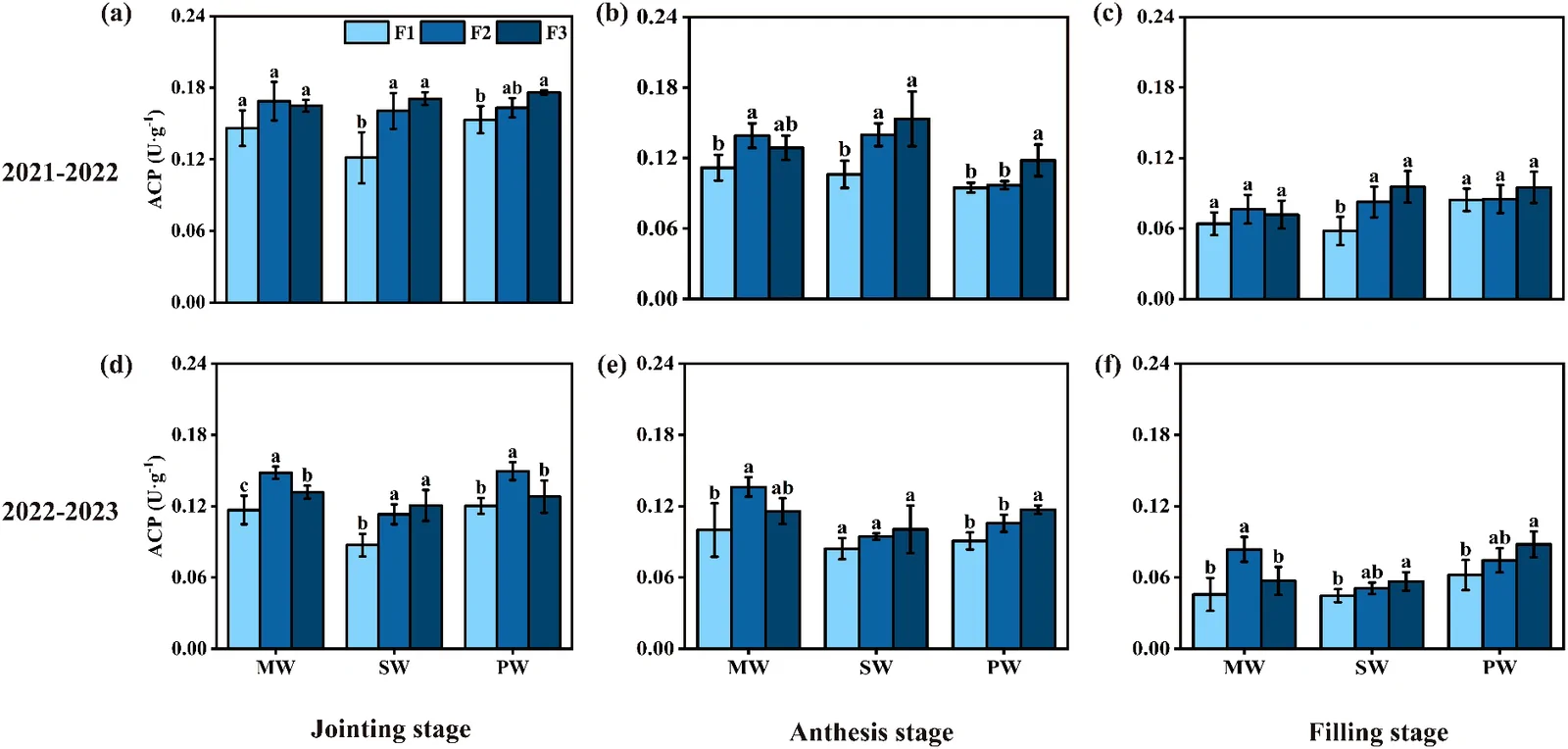
The ACP activity in wheat roots at different growth stages. MW: maize-wheat cropping system; SW: soybean-wheat cropping system; PW: peanut-wheat cropping system; F1: 0% organic substitution; F2: 25% chemical fertilizer substituted with organic fertilizer; F3: 50% chemical fertilizer substituted with organic fertilizer. ACP: acid phosphatase. The **(a–c)** means results at jointing, anthesis and filling stages in the 2021–2022 growing season, while **(d–f)** correspond to the three growth stages in the 2022–2023 growing season. Different lowercase letters indicate significant differences between treatments under the same cropping system (*p* < 0.05).

During the two years, the ACP activity gradually declined as wheat growth advanced. The ACP activity in wheat roots increased with organic substitution treatments compared with F1 (**Figure 6**). Under the MW cropping system, ACP exhibited the greatest increase in activity in F2, with an increase of 15.57%–82.77% compared with F1. Meanwhile, ACP showed a rise of 12.03%–24.72% in F3 compared with F1. Compared with F2, F3 decreased the activity of ACP (2.37%–31.76%). Meanwhile, under both SW and PW cropping systems, ACP activity increased with rising organic substitution rates. Compared with F1, F3 increased the ACP activity by 19.30%–64.90% under the SW cropping system and 6.53%–41.66% under the PW cropping system.

### 3.4 Nutrient accumulation and partitioning in wheat

**Figure 7:**
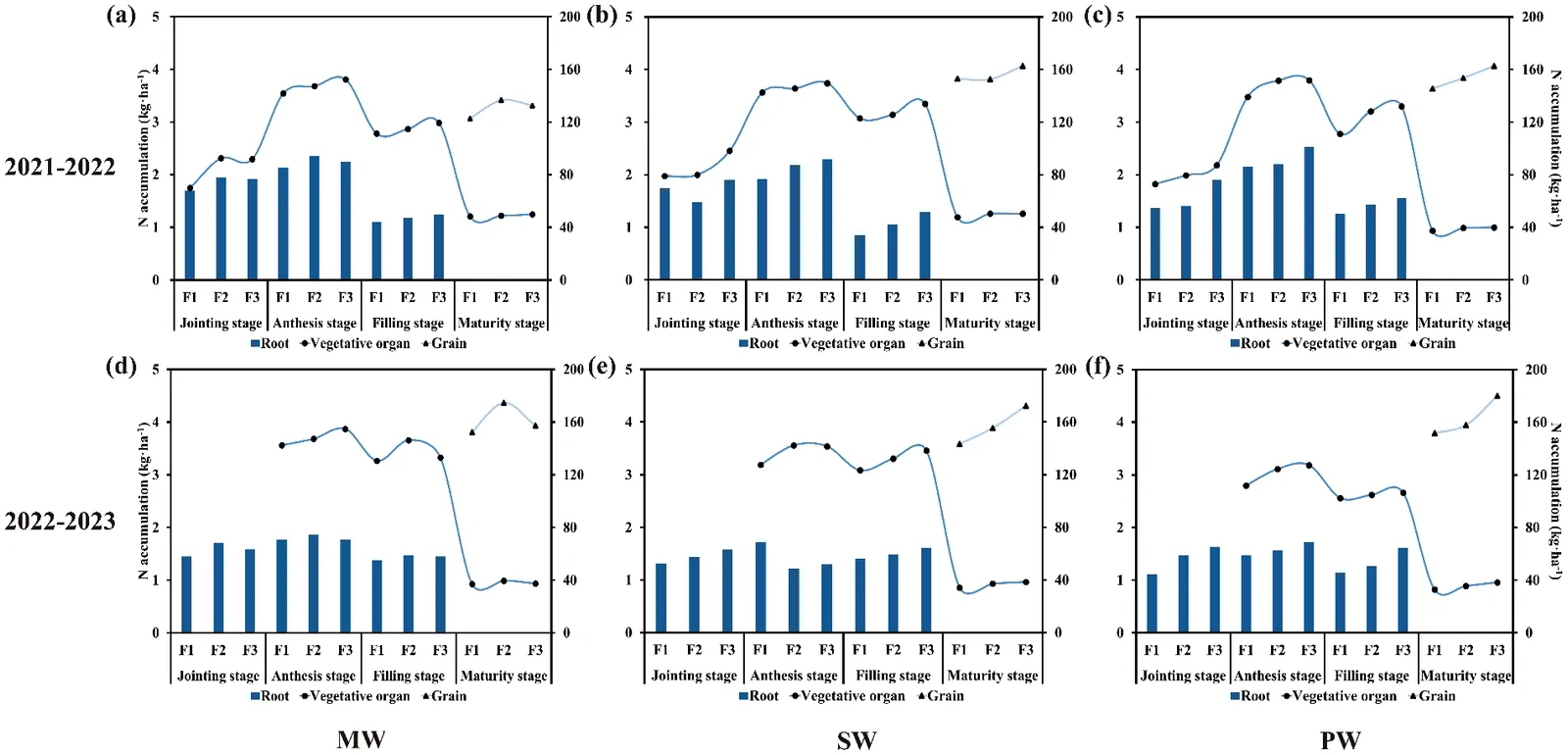
The effects of organic substitution on N uptake and accumulation in wheat under three cropping systems. MW: maize-wheat cropping system; SW: soybean-wheat cropping system; PW: peanut-wheat cropping system; F1: 0% organic substitution; F2: 25% chemical fertilizer substituted with organic fertilizer; F3: 50% chemical fertilizer substituted with organic fertilizer. The **(a–c)** means results at jointing, anthesis and filling stages in the 2021–2022 growing season, while **(d–f)** correspond to the three growth stages in the 2022–2023 growing season.

Further analysis showed that compared with F1, F2 increased the N accumulation under the MW cropping system, while F3 significantly increased the accumulation under SW and PW cropping systems (**Figure 7**). During the two years, the N accumulation in both the root and straw of wheat reached its peak at the anthesis stage, after which a gradual decline was observed. Under the MW cropping system, F2 increased the N accumulation by 6.00%–17.61% and 3.01%–13.87% in the wheat root and straw, and the wheat grain by 11.44%–14.61% compared to F1. Under SW and PW cropping systems, F3 increased N accumulation in the roots by 5.97%–28.75% and 2.33%–32.58%, respectively, compared to F1, and by 9.29%–52.69% and 16.57%–46.50%, respectively, compared to F2. Meanwhile, F3 increased N accumulation in straw by 4.95%–24.46% and 3.91%–19.66% compared to F1. Under the SW and PW cropping systems, F3 increased the N accumulation in grain by 6.39%–20.20% and 11.88%–18.54% compared to F1, and 6.69%–10.80% and 5.96%–13.99% compared to F2.

**Figure 8:**
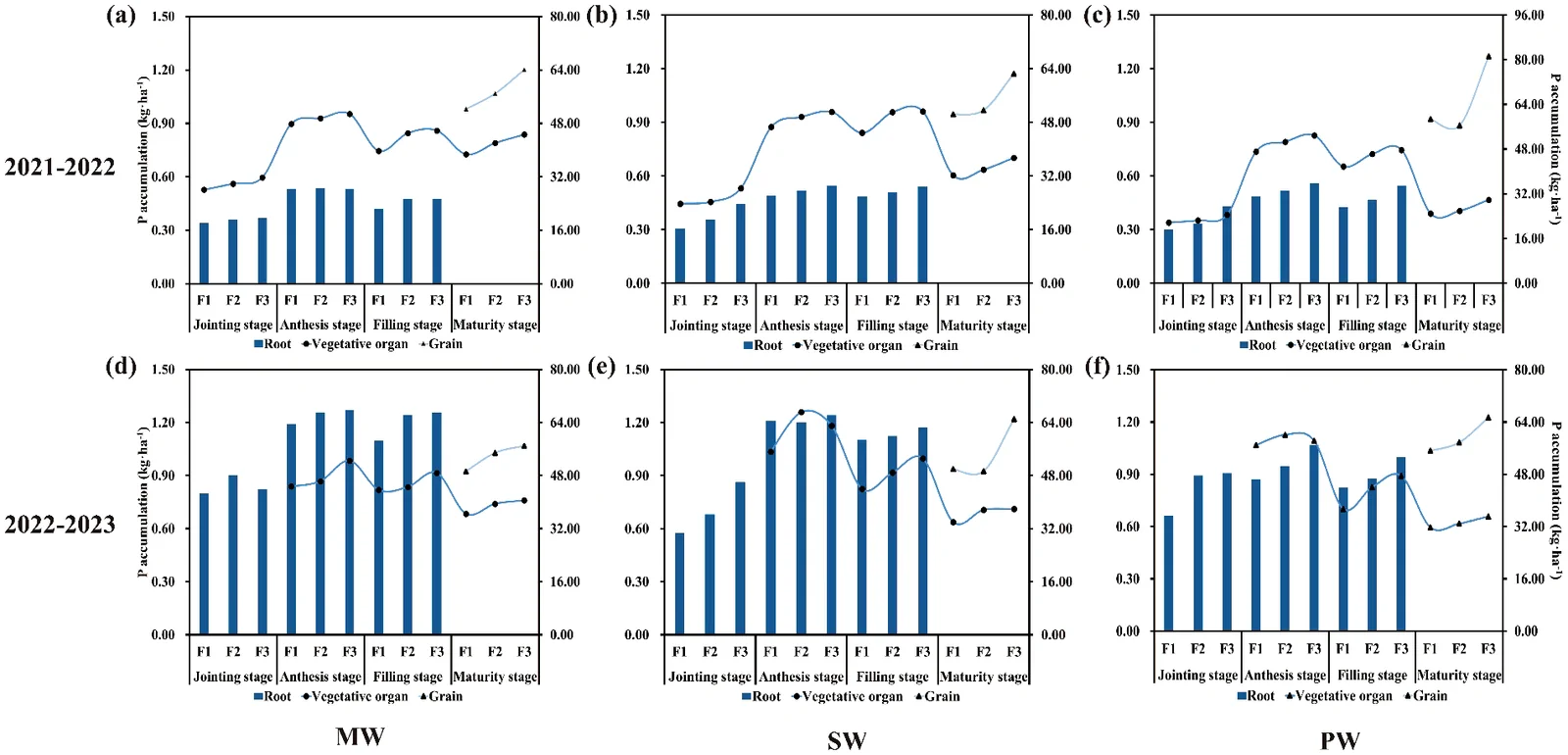
The effects of organic substitution on P uptake and accumulation in wheat under three cropping systems. MW: maize-wheat cropping system; SW: soybean-wheat cropping system; PW: peanut-wheat cropping system; F1: 0% organic substitution; F2: 25% chemical fertilizer substituted with organic fertilizer; F3: 50% chemical fertilizer substituted with organic fertilizer. The **(a–c)** means results at jointing, anthesis and filling stages in the 2021–2022 growing season, while **(d–f)** correspond to the three growth stages in the 2022–2023 growing season.

Compared with F1, organic fertilizer substitution increased the P accumulation in the tissues of wheat under three cropping systems (**Figure 8**). During the two years, P accumulation in both the root and straw of wheat reached its peak at the anthesis stage, after which a gradual decline was observed. Under the MW cropping system, P accumulation in wheat root and straw in F2 was significantly higher than in F1; F2 increased the accumulation of P in wheat grains by 8.84%–11.33% compared to F1. Under SW and PW cropping systems, F3 increased P accumulation in the roots by 0.48%–17.68% and 6.14%–35.04%, respectively, compared to F1 and 2.51%–12.04% and 3.12%–13.49%, respectively, compared to F2. Besides, F3 increased the P accumulation in straw compared to F1. Under SW and PW cropping systems, F3 increased P accumulation in grain by 24.07%–30.22% and 18.53%–38.22% compared to F1 and 21.11%–31.82% and 13.35%–44.00% compared to F2.

### 3.5 Nutrient-use efficiency of wheat

Organic substitution significantly improved N uptake and N-utilization efficiency of wheat across the three cropping systems (**Table 3**). Under the MW cropping system, F2 resulted in the highest NUE, NPFP, and NHI values. This treatment increased NUE, NPFP, and NHI by 8.60%–13.09%, 1.30%–5.41%, and 1.35%–2.63%, respectively, compared to F1. Meanwhile, F3 increased the NPFP and NHI values by 0.43%–0.65% and 0.41%–1.21%, respectively, compared to F1. Under the SW and the PW cropping systems, the F3 treatment resulted in the highest values of NUE, NPFP, and NHI; it increased NUE, NPFP, and NHI by 2.82%–10.66%, 1.73%–5.42%, and 0.13%–1.23%, respectively, compared to the F1. On the contrary, F2 decreased NPFP by 3.25%–11.55% and 0.78%–1.04% compared to F1 under SW and PW cropping systems, respectively.

**Table 3:** Effect of different organic substitution rates on N absorption and utilization of wheat under three cropping systems.

Year	Treatments	NUE (kg·kg^-1^)	NPFP (kg·kg^-1^)	NHI (%)
2021-2022	MWF1	0.76 ± 0.01b	44.95 ± 0.15b	71.77 ± 1.04b
	MWF2	0.82 ± 0.01a	47.38 ± 0.45a	73.66 ± 1.08a
	MWF3	0.81 ± 0.00a	45.24 ± 0.58b	72.64 ± 0.97b
	SWF1	0.89 ± 0.01b	47.87 ± 0.71b	76.25 ± 0.99b
	SWF2	0.90 ± 0.02b	46.31 ± 0.44c	75.14 ± 0.32c
	SWF3	0.95 ± 0.00a	50.47 ± 0.23a	76.35 ± 0.22a
	PWF1	0.81 ± 0.02b	46.51 ± 0.40ab	79.48 ± 0.70ab
	PWF2	0.86 ± 0.03a	46.15 ± 0.27b	79.50 ± 0.24b
	PWF3	0.90 ± 0.00a	46.98 ± 0.45a	80.25 ± 0.67a
2022-2023	MWF1	0.84 ± 0.01c	47.00 ± 0.63a	80.47 ± 0.11a
	MWF2	0.95 ± 0.00a	47.61 ± 0.14a	81.55 ± 0.06a
	MWF3	0.87 ± 0.01b	47.20 ± 0.86a	80.80 ± 0.50a
	SWF1	0.79 ± 0.01c	49.40 ± 0.44a	80.74 ± 0.76a
	SWF2	0.86 ± 0.04b	43.70 ± 0.37b	80.66 ± 0.73a
	SWF3	0.94 ± 0.01a	50.26 ± 0.80a	81.74 ± 0.68a
	PWF1	0.82 ± 0.01b	47.04 ± 0.19b	82.22 ± 0.49b
	PWF2	0.86 ± 0.03b	46.55 ± 0.95ab	81.61 ± 0.43ab
	PWF3	0.97 ± 0.01a	48.11 ± 0.63a	82.48 ± 0.11a
Treatment		*	*	*
Year		*	*	*
Treatment*Year		*	*	*

Means in a column for the same cropping system followed by different lowercase letters are significantly different (*p* < 0.05). *Mean significant difference at the 0.05 level.

Organic substitution significantly improved P uptake and utilization efficiency in wheat across the three cropping systems (**Table 4**). Under the MW cropping system, PUE and PHI increased with rising proportions of organic substitution. The F2 treatments resulted in the highest PPFP value; it increased PPFP by 1.30%–5.41% and 0.86%–4.51% compared to F1 and F3, respectively. In both the SW and the PW cropping systems, PUE, PPFP, and PHI were the highest in the F3 treatment. The F2 treatment decreased PPFP and PHI by 3.25%–11.55% and 1.02%–4.62%, respectively, compared to F1 under the SW cropping system, and PPFP by 0.78%–1.04% compared to F1 under the PW cropping system.

**Table 4:** Effect of different organic substitution rates on P absorption and utilization of wheat under three cropping systems.

Year	Treatments	PUE (kg/kg)	PPFP (kg/kg)	PHI (%)
2021-2022	MWF1	0.61 ± 0.01c	84.28 ± 0.29b	57.44 ± 0.36b
	MWF2	0.66 ± 0.01b	88.84 ± 0.84a	57.48 ± 0.15b
	MWF3	0.72 ± 0.02a	84.83 ± 1.08b	58.93 ± 0.54a
	SWF1	0.55 ± 0.01c	89.76 ± 1.33b	60.98 ± 1.19a
	SWF2	0.57 ± 0.00b	86.84 ± 0.83c	60.36 ± 0.17a
	SWF3	0.67 ± 0.02a	94.63 ± 0.43a	62.52 ± 1.22a
	PWF1	0.56 ± 0.02c	87.22 ± 0.75ab	70.19 ± 1.14b
	PWF2	0.55 ± 0.01b	86.53 ± 0.51b	68.55 ± 1.44b
	PWF3	0.74 ± 0.02a	88.09 ± 0.84a	73.14 ± 0.60a
2022-2023	MWF1	0.57 ± 0.02b	88.12 ± 1.18a	57.46 ± 1.61a
	MWF2	0.63 ± 0.01a	89.27 ± 0.26a	58.15 ± 0.51a
	MWF3	0.65 ± 0.01a	88.50 ± 1.60a	58.43 ± 0.77a
	SWF1	0.56 ± 0.03b	92.63 ± 0.83a	59.45 ± 3.78a
	SWF2	0.58 ± 0.01b	81.93 ± 0.70a	56.71 ± 1.64a
	SWF3	0.69 ± 0.01a	94.23 ± 1.50a	63.17 ± 0.88a
	PWF1	0.58 ± 0.02b	88.20 ± 0.35ab	63.46 ± 1.66a
	PWF2	0.60 ± 0.01b	87.28 ± 1.78b	63.69 ± 1.05a
	PWF3	0.67 ± 0.02a	90.20 ± 1.18a*	65.06 ± 1.61a
Treatment	*	*	*	*
Year	*	*	*	*
Treatment*Year	*	*	*	*

Means in a column for the same cropping system followed by different lowercase letters are significantly different (*p* < 0.05). *Mean significant difference at the 0.05 level.

### 3.6 Comprehensive analysis of factors affecting wheat yield

Our analyses showed that F2 yielded the most favorable outcomes under the MW cropping system, whereas F3 performed best under the SW and the PW cropping systems. Subsequently, RF and SEM analyses were conducted to elucidate the key factors and pathways associated with the effects of organic substitution on wheat yield across different planting systems. The RF model identified TN content in wheat roots as the major influencing factor, indicating that N accumulation plays a dominant role in determining wheat grain yield under the MW cropping system (**Supplementary Figure 5D**). Under the SW and PW cropping systems, P-acq was identified as the most critical indicator (**Supplementary Figures 5E, F**). Among them, the importance of multiple indicators, such as AP, N_min_, and NUE, under the SW cropping system was relatively balanced, while the dominance of P-related indicators, such as P-acq, TP, and PHI, under the PW cropping system was pronounced. Additionally, N_min_ also considerably contributed.

SEM analysis showed that across the three cropping systems, organic substitution promoted the activities of N- and P-metabolic enzymes in wheat roots, the accumulation of root nutrients, and the contribution rate of pre-anthesis nutrition translocation to grain by directly influencing the activities of rhizosphere soil N-acq/P-acq enzymes and soil nutrient contents. In addition, a relationship was identified between the metabolic enzyme activities in wheat roots and rhizosphere soil.

## 4 Discussion

### 4.1 Nutrient-acquiring enzymes and nutrition availability in the rhizosphere soil

Studies have shown that the application of organic fertilizer increases the activity of soil N-acq enzymes and the content of N_min_ (Lu et al., 2023; Wang et al., 2024b; Yang et al., 2024). Previous conclusions were largely based on experimental designs with inconsistent total fertilizer application rates, whereas this study adopted an equal total fertilizer input scheme. In our study, partial substitution of chemical fertilizer with organic fertilizer in a wheat field under three cropping systems decreased rhizosphere soil N-acq enzyme activity and N_min_ content, but increased P-acq enzyme activity and AP content during the two years (**Figures 3**-**6**). Partial manure substitution facilitated the proliferation of soil microorganisms (Gao et al., 2023). Consequently, during the early stage of crop growth, microorganisms immobilized a greater amount of soil N_min_, which was gradually released during the middle and late stages of growth (Gao et al., 2023; Nyameasem et al., 2021; Ullah et al., 2021). Compared with F2, the F3 treatment increased rhizosphere soil N-acq enzyme activity and N_min_ content under three cropping systems in our study (**Figures 1**, **3**). Organic fertilizers primarily supply organic N, which is gradually converted to inorganic N, further reducing the size of the unstable N pool (de Figueiredo et al., 2018; Wang et al., 2020). Generally, at low substitution rates, the organic N pool is relatively small, and N immobilization predominates, thereby limiting the release of inorganic N through mineralization (Tang et al., 2022). However, at moderate substitution rates, the organic N pool expands significantly, accompanied by increased C and N substrate availability, enhanced microbial activity, and accelerated organic N mineralization. As a result, the total amount of inorganic N released surpasses the quantity immobilized due to C-induced N fixation (Ning et al., 2022; Rezig et al., 2013). This is consistent with the study’s conclusion.

**Figure 9:**
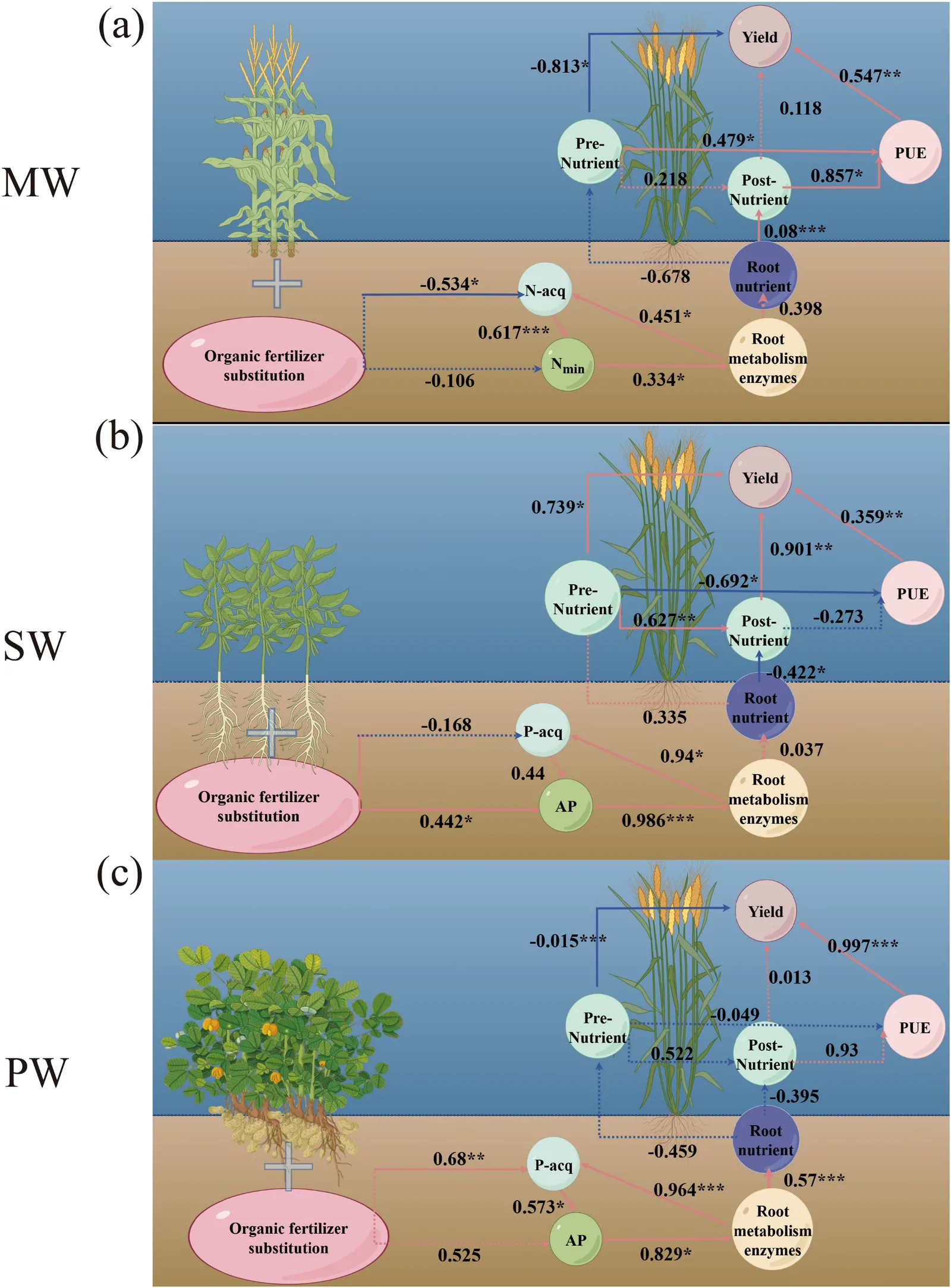
The structural equation model (SEM) evaluated the effects of organic substitution on rhizosphere soil N and P transformation, plant nutrient uptake and utilization, and grain yield in wheat. MW: maize-wheat cropping system **(a)**; SW: soybean-wheat cropping system **(b)**; PW: peanut-wheat cropping system **(c)**. TN: total N accumulation of wheat root; TP: total P accumulation of wheat root; pre-N: contribution rate of pre-anthesis N translocation amount to grain; pre-P: contribution rate of pre-anthesis P translocation amount to grain; post-N: contribution rate of pre-anthesis N translocation amount to grain; post-P: contribution rate of pre-anthesis P translocation amount to grain. Red and blue lines represent positive and negative paths, respectively. Solid lines represent significance, while dashed lines represent non-significance <. Asterisks ‘*’, ‘**’ and ‘***’ indicate significance at p < 0.05, p < 0.01 and p < 0.001 levels, respectively. MW: CMIN/DF = 2.855; SW: CMIN/DF = 2.956; PW: CMIN/DF = 2.747.

In contrast to the N, P availability exhibited a divergent pattern under organic substitution. A study on the maize-cabbage rotation system demonstrated that organic substitution for chemical fertilizers improved rhizosphere soil P availability (Qiao et al., 2019), and a meta-analysis showed that organic manure plus chemical fertilizer results in increased soil phosphatase activity (Miao et al., 2019). Similarly, in the field, the soil AP content was higher under organic fertilization than under chemical fertilization and increased further with rising organic fertilizer substitution ratios. There is a positive correlation between APase activity in root secretions and within the roots (Wang et al., 2024a). Root-secreted phosphatase and phytase predominantly regulate their activities directly in rhizosphere soil (Jung et al., 2018; Liang et al., 2014). The present study found that the application of organic fertilizer increased ACP activity in wheat roots under the three cropping systems, thereby accelerating the conversion of organic P to inorganic P and increasing soil AP content. The SEM similarly revealed (**Figure 9**).

### 4.2 Crop growth and nutrition utilization

Soil fertility reflects soil fertility and are essential indicators of crop growth and yield formation. Previous studies have demonstrated that the addition of organic fertilizers, combined with a reduction in inorganic fertilizer input, increases rhizosphere soil nutrients and promotes crop root growth (Ren et al., 2021). In this study, partial substitution in three cropping systems increased the activity of enzymes associated with N and P metabolism in the wheat roots. Here, F2 treatment resulted in the highest activity of metabolic enzymes in wheat roots under the MW cropping system, while F3 resulted in the highest activity under the SW and the PW cropping systems (**Figures 5**, **6**; **Supplementary Figures 2**–**4**). The optimal concentration and chemical forms of soil N and P under this substitution regime enhanced root nutrition uptake. Additionally, the nutrients and beneficial microorganisms in organic fertilizers contribute to improved root development and nutrient acquisition (Li et al., 2024; Wahidurromdloni et al., 2025). Moreover, the bioactive components in organic fertilizers stimulate N metabolism-related enzymes in wheat, thereby promoting N assimilation and improving NUE (Li et al., 2024; Yuan et al., 2023). Research has indicated that when organic fertilizers are applied, organic P is gradually released from the fertilizers, stimulating plant roots to secrete acid phosphatase; this change in turn promotes the release of inorganic P for uptake by plant roots (Wang et al., 2026).

At the whole-plant level, our study showed that partial organic fertilizer substitution in wheat across three cropping systems improved nutrient utilization by promoting post-anthesis nutrient accumulation (**Figures 7**, **8**). This is due to the water-retention and slow-release nutrient characteristics of manure (Xie et al., 2017). Moreover, partial substitution of chemical fertilizer with organic fertilizer delayed leaf senescence during the mid-to-late stage of grain filling, thereby increasing nutrient uptake and yield (He et al., 2022). Combined organic and chemical fertilizer exploits the complementary fertilizer characteristics of both inputs, ensuring nutrient supply and prevents nutrient loss during the growing season, promoting crop nutrient uptake and thereby enhancing nutrient-use efficiency and crop yield (Gao et al., 2023; Wang et al., 2020). Furthermore, the N and P in organic fertilizers exist in diverse chemical forms. When applied in combination with chemical fertilizers, organic fertilizers provide multiple nutrient sources and enhance N and P uptake and translocation in crops (Makino et al., 2022; Sun et al., 2024).

It should be noted that the relationship between substitution rate and nutrient use efficiency is not always linear. Qiang et al. (2025) indicated that 35% organic fertilizer substitution rate significantly increased the NUE and PUE of wheat compared to the control treatment, favor while NUE declined at 50% substitution. Several other researchers have documented that the application of organic fertilizer in combination with inorganic fertilizer reduces N and P uptake efficiency in double rice cropping systems (Qaswar et al., 2020). A 70% substitution of chemical fertilizers with manure improved PUE and increased rice yield and crop P uptake (Hayatu et al., 2023). Compared with the sole application of inorganic fertilizer, a combination of inorganic and organic fertilizers decreased PUE in both the wheat–fallow and wheat–maize cropping systems (Khan et al., 2018). These differences among studies may be attributed to cropping systems, soil types, and climatic conditions, which influence the mineralization of organic manure and, consequently, nutrient supply and uptake (Liu et al., 2021b).

### 4.3 Grain yield

Stable or high crop production is a key criterion for evaluating agricultural management practices (Yang et al., 2017). Under the maize-wheat and soybean-wheat cropping systems, the integrated application of organic and inorganic fertilizers positively affects soil quality and enhances wheat yield (Song et al., 2022). A previous study reported that 45% of organic fertilizer alternatives enhanced crop yields and grain quality by improving soil quality in the maize-wheat cropping system (Wu et al., 2024a). In contrast, another study indicated that a 20% organic fertilizer substitution in the maize-wheat cropping system maintains crop yields, enhances soil fertility, and concurrently reduces human health risks (Liu et al., 2023b). Ma et al. (2025) demonstrated that 30% organic fertilizer substitution rate did not significantly increase wheat yield in the maize-wheat and soybean-wheat cropping systems. In our study, partial organic fertilizer substitution increased grain yield under MW cropping systems, while the F3 treatment increased yield under the PW and SW cropping system (**Table 2**). The increase in crop yields under partial organic fertilizer substitution may be attributed to simultaneous improvement in nutrient availability and soil fertility (Tang et al., 2022), as well as enhanced N and P agronomic efficiency (Li et al., 2022). The underlying mechanism is consistent with Liu et al. (2021a), who indicated that the increase in yield from the combined application of organic and inorganic fertilizers is due to enhanced nutrient uptake and physiological use efficiency, which is consistent with our findings. This improved efficiency facilitates the conversion of N into photosynthetic products, thereby promoting plant growth and grain yield.

However, the yield response was system- and rate-dependent. Under the SW and PW cropping systems, the wheat yield in the F2 treatment decreased compared with F1. Although 25% substitution led to an increase in soil nutrients and plant nutrient accumulation, the translocation rates of N and P decreased (**Supplementary Tables 1**, **2**). Consequently, a substantial quantity of nutrients was retained in vegetative organs such as stems and leaves rather than being allocated to the grains. Meanwhile, in the F2 treatment, although the number of spikes increased, both the number of grains per spike and the 1000-grain weight decreased. SEM results further showed that the contribution rate of post-anthesis nutrient accumulation to grain and yield is positively correlated (**Figure 9**), explaining why the reduced translocation efficiency under F2 translated into lower yields in SW and PW.

The differential yield responses across cropping systems also clarify why legume-based rotations required distinct optimal substitution rates compared to the MW system. The low C/N of legume residues leads to rapid N mineralization and a lack of N release uptake synchrony with the subsequent cash crop, resulting in N losses that must be compensated by additional organic inputs (Diacono et al., 2019; Pang and Letey, 2000). Moreover, low C/N legume residues contribute less to stable SOC formation than high C/N cereal residues, so legume-based systems rely more heavily on high C/N manures to maintain SOM reserves and soil structure (Alemayehu et al., 2020). These effects raise the required organic substitution rate in legume-based rotations relative to a maize–wheat cropping system, where cereal straw itself supplies a substantial high C/N organic input and N release is slower but more synchronous with management applications (Liu et al., 2025).

Maintaining yield stability is becoming increasingly important to maintain food security (Grassini et al., 2013). A meta-analysis demonstrated that organic substitution increased wheat yield by 3.3% (Ren et al., 2023). 30%–50% organic substitution could improve wheat yield by 4.2%–9.2% (Yang et al., 2021). Another study has reported yield reduction under organic fertilizer substitution (Chander et al., 2023). In the present study, compared with the non-substitution control, the 25% organic substitution only significantly increased grain yield under the MW cropping system, and the 50% organic substitution significantly increased grain yield under the SW cropping system in 2021–2022. organic substitution stabilized grain yield, promoted soil nutrient transformation, and improved nutrient use efficiency compared with the control. Future research will further investigate the long-term effects of cropping regimes and organic substitution.

## 5 Conclusions

This study proved that organic fertilizer substitution significantly reduced rhizosphere soil N-acq enzyme activity and N_min_ content, but increased P-acq enzyme activity and AP content. It enhanced the activities of enzymes related to N and P metabolism in wheat roots, thereby promoting N and P accumulation. Within the three tested substitution rates, the 25% organic fertilizer substitution yielded the most favorable outcomes under the maize-wheat cropping system, while under the soybean-wheat and peanut-wheat cropping systems, the 50% substitution ratio demonstrated superior performance. Furthermore, indicators related to N transformation and utilization were identified as the key drivers of yield formation under the maize-wheat cropping system, whereas those related to P transformation and utilization were identified as the key drivers of yield formation under the soybean-wheat and the peanut-wheat cropping systems. In conclusion, the study proved that optimizing N- and P-use efficiencies and achieving high and stable wheat yields require specific cropping systems with appropriate organic fertilizer substitution ratios. Thus, the study’s findings provide a scientific basis for optimizing N and P management in wheat cultivation based on the cropping system and region. However, future research should focus on the long-term effects of rational organic fertilization regimes across diverse cropping systems, while further exploring their corresponding environmental implications including nutrient losses and greenhouse gas emissions.

## Data Availability

The raw data supporting the conclusions of this article will be made available by the authors, without undue reservation.

## Supplementary materials

Supplementary Figure 1 Monthly average temperature and monthly precipitation during the experimental growing seasons. **(a)**: monthly average temperature; **(b)**: monthly precipitation.

Supplementary Figure 2 The GOGAT activities in wheat root at different growth stages. MW: maize-wheat cropping system; SW: soybean-wheat cropping system; PW: peanut-wheat cropping system; F1: 0% organic substitution; F2: 25% chemical fertilizer substituted with organic fertilizer; F3: 50% chemical fertilizer substituted with organic fertilizer. GOGAT: glutamate synthase-glutamine oxidoreductase. The **(a–c)** means results at jointing, anthesis and filling stages in the 2021–2022 growing season, while **(d–f)** correspond to the three growth stages in the 2022–2023 growing season. Different lowercase letters indicate significant differences between treatments under the same cropping system (*p* < 0.05).

Supplementary Figure 3 The GDH activities in wheat roots at different growth stages. MW: maize-wheat cropping system; SW: soybean-wheat cropping system; PW: peanut-wheat cropping system; F1: 0% organic substitution; F2: 25% chemical fertilizer substituted with organic fertilizer; F3: 50% chemical fertilizer substituted with organic fertilizer. GDH: glutamate dehydrogenase. The **(a–c)** means results at jointing, anthesis and filling stages in the 2021–2022 growing season, while **(d–f)** correspond to the three growth stages in the 2022–2023 growing season. Different lowercase letters indicate significant differences between treatments under the same cropping system (*p* < 0.05).

Supplementary Figure 4 The GS activities in wheat roots at different growth stages. MW: maize-wheat; SW: soybean-wheat; PW: peanut-wheat; F1: 0% organic substitution of chemical fertilizer; F2: 25% organic substitution of chemical fertilizer; F3: 50% organic substitution of chemical. GS: glutamine synthetase. The **(a–c)** means results at jointing, anthesis and filling stages in the 2021–2022 growing season, while **(d–f)** correspond to the three growth stages in the 2022–2023 growing season. Different lower-case letters indicate that differences are significant under the same crop rotation system (*p* < 0.05).

Supplementary Figure 5 Correlation analysis among various indicators **(A–C)** and random forest analysis of wheat yield **(D–F)**. MW: maize-wheat; SW: soybean-wheat; PW: peanut-wheat. TN: N accumulation of wheat root; TP: P accumulation of wheat root; pre-N: contribution rate of pre-anthesis N translocation amount to grain; pre-N: contribution rate of pre-anthesis N translocation amount to grain; pre-P: contribution rate of pre-anthesis P translocation amount to grain; post-N: contribution rate of pre-anthesis N translocation amount to grain; post-P: contribution rate of pre-anthesis P translocation amount to grain.

Supplementary Table 1 Effect of different organic substitution rate on N transport of aboveground parts and its contribution rate to N accumulation in grain of wheat under three cropping systems. Means in a column for the same crop followed by different lowercase letters indicate a significant difference at *p* < 0.05. *means significant difference at the 0.05 level.

Supplementary Table 2 Effect of different organic substitution rate on P transport of aboveground parts and its contribution rate to P accumulation in grain of wheat under three cropping systems. Means in a column for the same crop followed by different lowercase letters indicate a significant difference at *p* < 0.05. *means significant difference at the 0.05 level.
