# Supplementary material for: Cropping system regulates the optimal organic substitution ratio via soil-plant nitrogen and phosphorus transformation and utilization
Source: Front Plant Sci. 2026 Aug 6;17:1877524. doi: 10.3389/fpls.2026.1877524 (PMC13492241; doi:10.3389/fpls.2026.1877524)
Supplement: Supplementary file 1 [file Supplementaryfile1.pdf]

Supplementary figures and tables

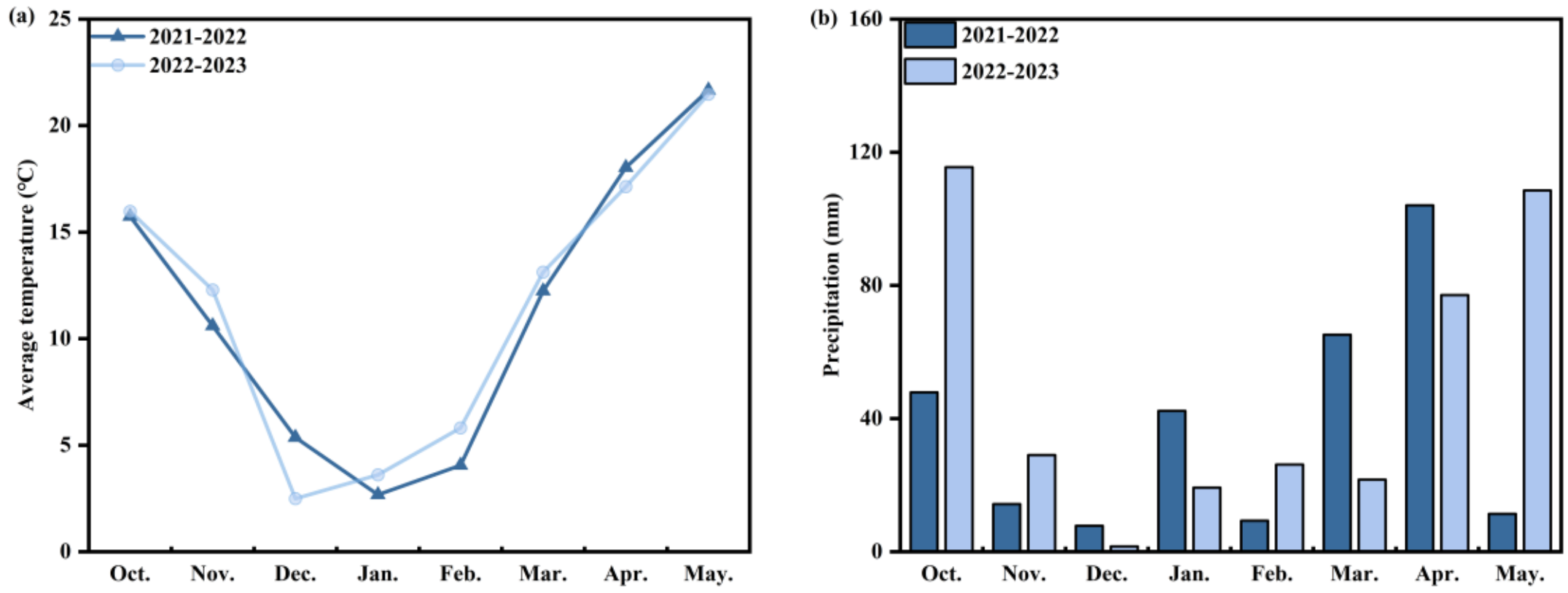

Fig. S1 Monthly average temperature and monthly precipitation during the experimental growing seasons. a: monthly average temperature; b: monthly precipitation.

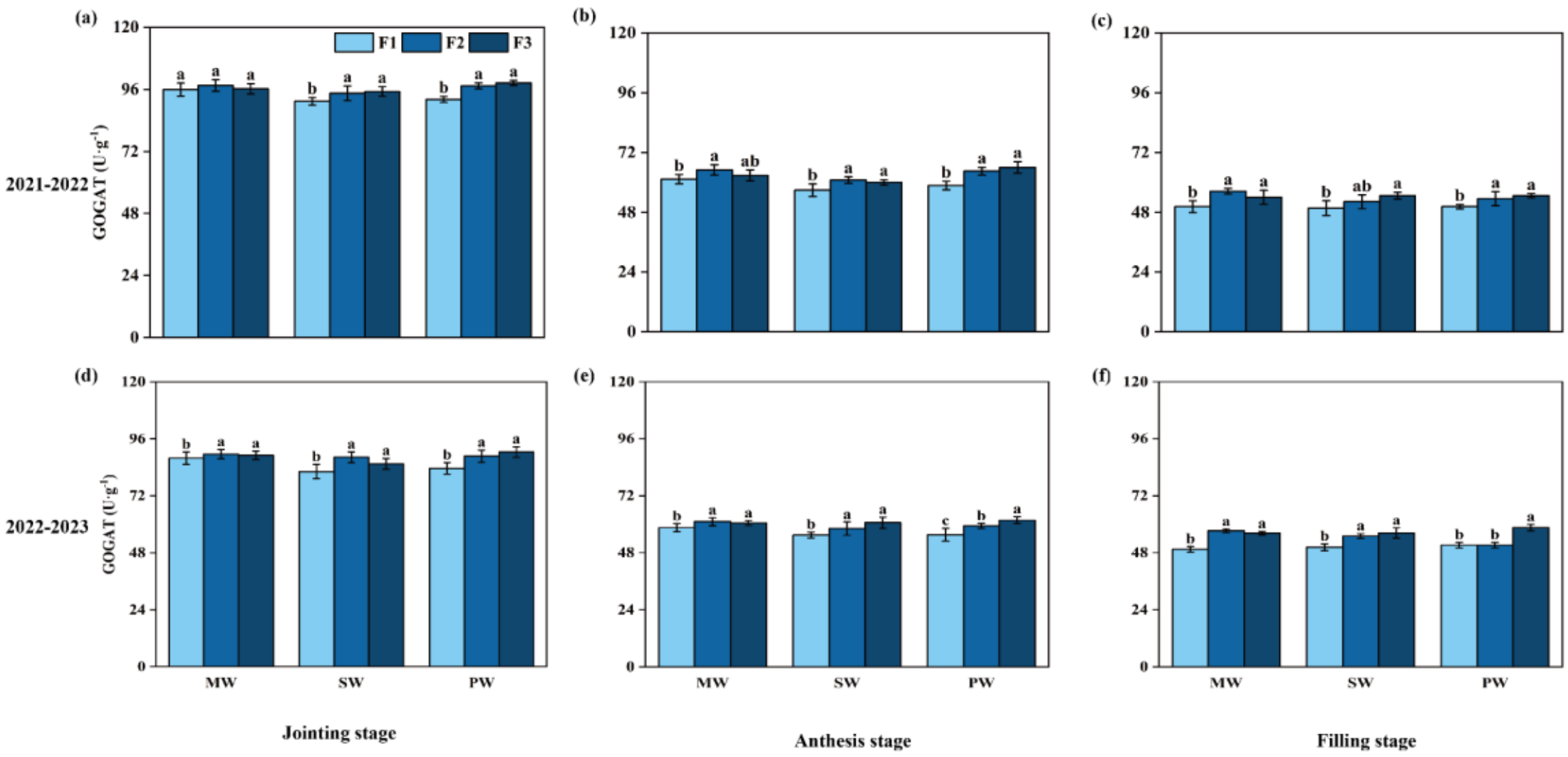

Fig. S2 The GOGAT activities in wheat root at different growth stages. MW: maize-wheat cropping system; SW: soybean-wheat cropping system; PW: peanut-wheat cropping system; F1: 0% organic substitution; F2: 25% chemical fertilizer substituted with organic fertilizer; F3: 50% chemical fertilizer substituted with organic fertilizer. GOGAT: glutamate synthase-glutamine oxidoreductase. The a, b, c means results at jointing, anthesis and filling stages in the 2021–2022 growing season, while d, e, f correspond to the three growth stages in the 2022–2023 growing season. Different lowercase letters indicate significant differences between treatments under the same cropping system ($p < 0.05$).

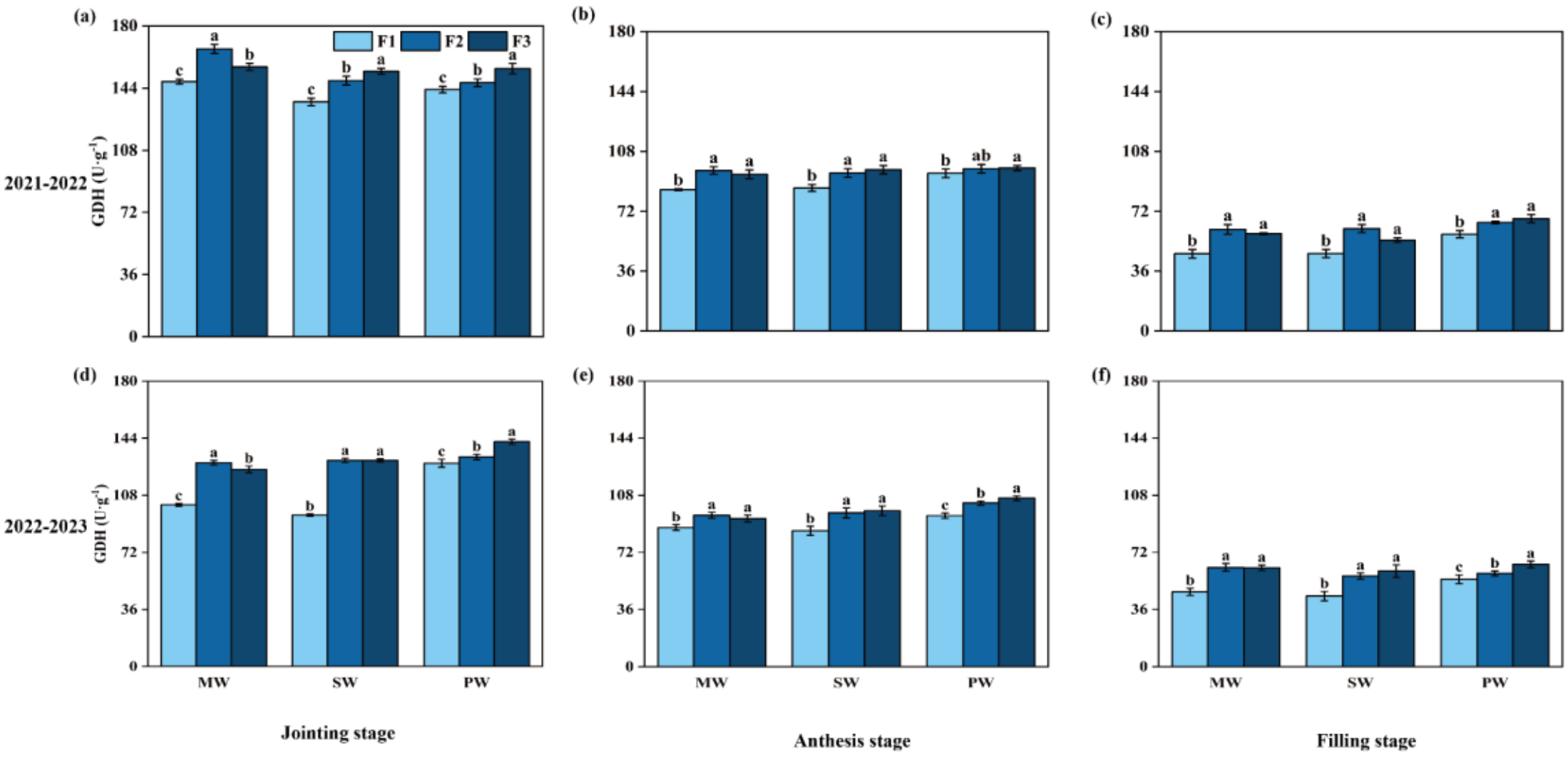

Fig. S3 The GDH activities in wheat roots at different growth stages. MW: maize-wheat cropping system; SW: soybean-wheat cropping system; PW: peanut-wheat cropping system; F1: 0% organic substitution; F2: 25% chemical fertilizer substituted with organic fertilizer; F3: 50% chemical fertilizer substituted with organic fertilizer. GDH: glutamate dehydrogenase. The a, b, c means results at jointing, anthesis and filling stages in the 2021–2022 growing season, while d, e, f correspond to the three growth stages in the 2022–2023 growing season. Different lowercase letters indicate significant differences between treatments under the same cropping system ($p < 0.05$).

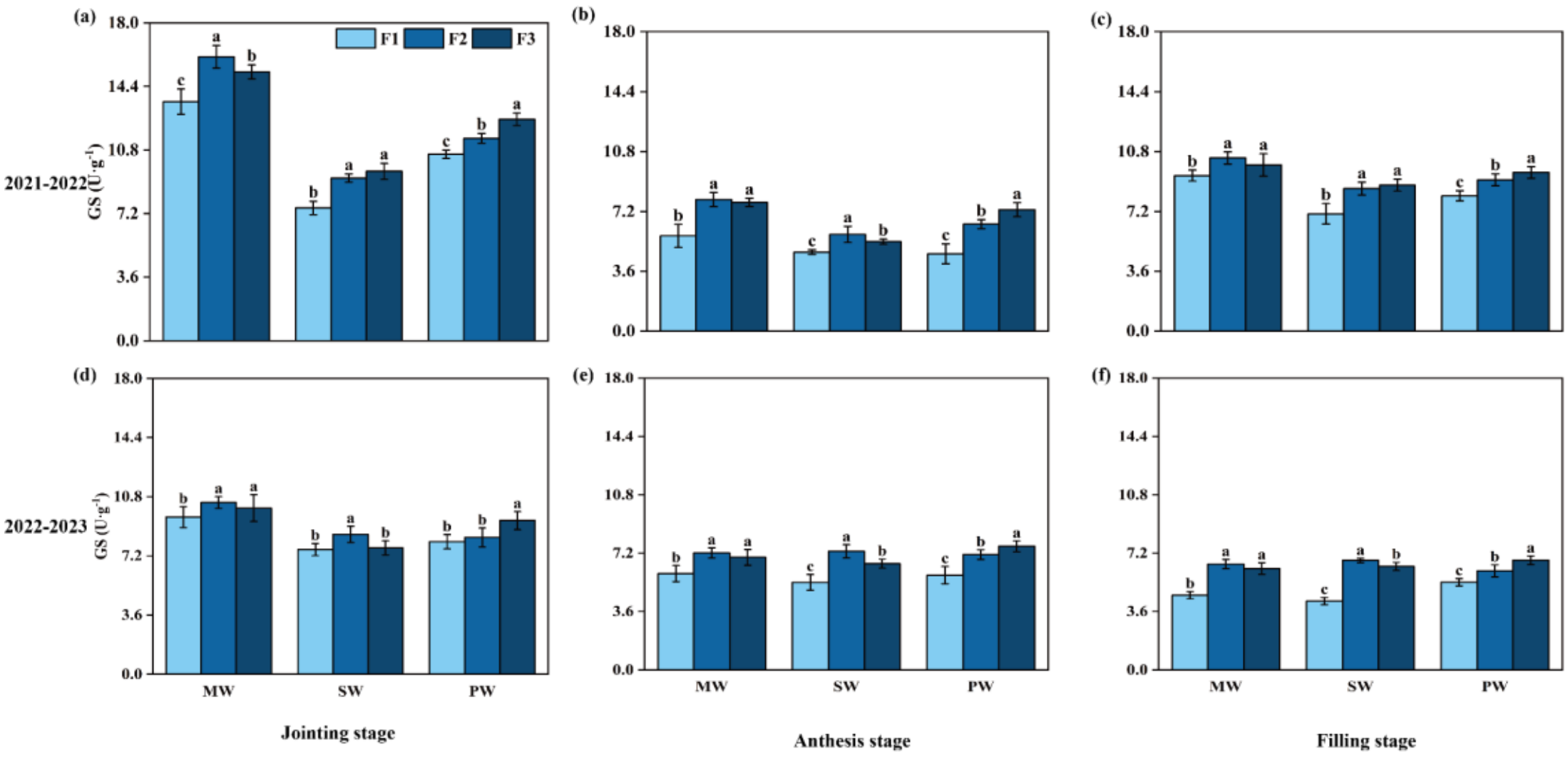

Fig. S4 The GS activities in wheat roots at different growth stages. MW: maize-wheat; SW: soybean-wheat; PW: peanut-wheat; F1: 0% organic substitution of chemical fertilizer; F2: 25% organic substitution of chemical fertilizer; F3: 50% organic substitution of chemical. GS: glutamine synthetase. The a, b, c means results at jointing, anthesis and filling stages in the 2021–2022 growing season, while d, e, f correspond to the three growth stages in the 2022–2023 growing season. Different lower-case letters indicate that differences are significant under the same crop rotation system ($p < 0.05$).

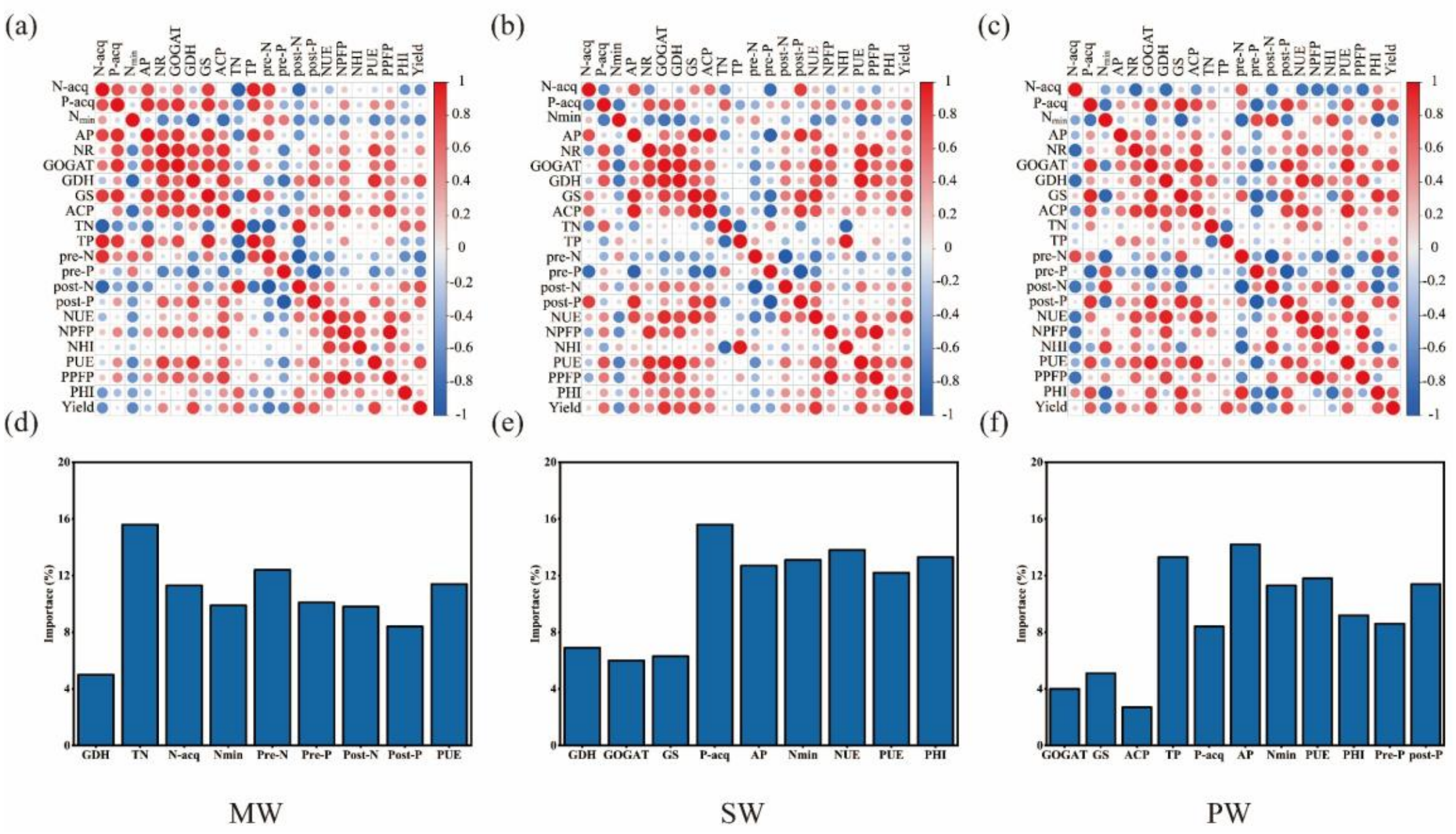

Fig. S5 Correlation analysis among various indicators (a, b, c) and random forest analysis of wheat yield (d, e, f). MW: maize-wheat; SW: soybean-wheat; PW: peanut-wheat. TN: N accumulation of wheat root; TP: P accumulation of wheat root; pre-N: contribution rate of pre-anthesis N translocation amount to grain; pre-N: contribution rate of pre-anthesis N translocation amount to grain; pre-P: contribution rate of pre-anthesis P translocation amount to grain; post-N: contribution rate of pre-anthesis N translocation amount to grain; post-P: contribution rate of pre-anthesis P translocation amount to grain.

Table S1 Effect of different organic substitution rate on N transport of aboveground parts and its contribution rate to N accumulation in grain of wheat under three cropping systems

Year	Treatment	Pre-anthesis N translocation amount ($kg\cdot ha^{-1}$)	Pre-anthesis N transport rate (%)	Contribution rate of pre-anthesis N translocation amount to grain (%)	Post-anthesis N accumulation amount (kg ha^{-1})	Contribution rate of post-anthesis N accumulation amount to grain (%)
2021-2022	MWF1	93.45±2.50a	65.95±1.60a	76.19±1.63a	29.19±1.90b	23.81±1.63a
	MWF2	98.33±2.26a	66.79±1.59a	71.95±1.58a	38.34±2.11a	28.05±1.58a
	MWF3	102.35±7.14a	67.15±2.37a	77.14±4.42a	30.26±5.44b	22.86±4.42a
	SWF1	94.94±3.91a	66.56±2.03a	62.06±3.08a	58.09±5.33a	37.94±3.08a
	SWF2	95.11±2.24a	65.32±0.78a	62.36±2.50a	57.49±5.14a	37.64±2.50a
	SWF3	99.24±3.66a	66.30±0.86a	60.96±2.29a	63.56±3.82a	39.04±2.29a
	PWF1	101.59±2.65b	73.01±1.45a	69.93±3.11a	43.78±5.60ab	30.07±3.11a
	PWF2	112.02±3.04a	73.89±0.75a	73.01±2.01a	41.47±3.91b	26.99±2.01a
	PWF3	111.73±1.83a	73.62±0.94a	68.70±0.47a	50.91±0.28a	31.30±0.47a
2022-2023	MWF1	105.45±0.76b	74.03±0.56b	69.21±1.49b	46.95±2.98b	30.79±1.49b

	MWF2	107.74±1.37b	73.16±0.23b	61.69±0.72c	66.92±1.24a	38.31±0.72a
	MWF3	117.30±2.22a	75.81±0.80a	74.50±0.85a	40.15±1.40c	25.50±0.85c
	SWF1	93.30±3.80b	73.16±1.72a	65.12±3.17ab	50.00±4.94b	34.88±3.17ab
	SWF2	105.11±7.03a	73.80±1.49a	67.66±4.34a	50.35±7.84b	32.34±4.34b
	SWF3	103.10±3.07ab	72.81±1.18a	59.86±1.75b	69.15±3.44a	40.14±1.75a
	PWF1	79.02±3.32b	70.63±1.57a	52.07±2.79ab	72.81±5.08b	47.93±2.79ab
	PWF2	88.84±3.23a	71.39±1.62a	56.37±4.06a	69.05±9.01b	43.63±4.06b
	PWF3	89.11±0.63a	69.97±0.39a	49.51±0.62b	90.87±2.14a	50.49±0.62a
Treatment		*	*	*	*	*
Year		*	*	*	*	*
Treatment*Year		*	*	*	*	*

Means in a column for the same crop followed by different lowercase letters indicate a significant difference at $p <$ 0.05. * means significant difference at the 0.05 level.

Table S2 Effect of different organic substitution rate on P transport of aboveground parts and its contribution rate to P accumulation in grain of wheat under three cropping systems

Year	Treatment	Pre-anthesis P translocation amount ($kg \cdot ha^{-1}$)	Pre-anthesis P transport rate (%)	Contribution rate of pre-anthesis nutrition translocation amount to grain (%)	Post-anthesis P accumulation amount (kg ha^{-1})	Contribution rate of post-anthesis P accumulation amount to grain (%)
2021-2022	MWF1	9.14±0.99a	19.07±1.60a	17.48±1.78a	43.15±1.32c	82.52±1.78b
	MWF2	6.84±2.23a	13.87±3.83b	12.01±3.83ab	50.07±2.02b	87.99±3.83ab
	MWF3	6.15±2.12a	12.02±3.58b	9.62±3.37b	57.91±2.63a	90.38±3.37a
	SWF1	14.39±2.78a	30.73±4.12a	28.47±4.47a	35.98±1.21b	71.53±4.47b
	SWF2	15.74±0.94a	31.70±1.25a	30.50±1.93a	35.87±1.12b	69.50±1.93b
	SWF3	13.66±0.24a	26.74±0.57a	21.89±1.13b	48.83±2.88a	78.11±1.13a
	PWF1	22.15±1.25b	47.01±1.27a	37.78±3.82b	36.66±4.11b	62.22±3.82b
	PWF2	24.73±1.49ab	48.85±1.80a	43.83±2.60a	31.72±2.27b	56.17±2.60c
	PWF3	23.05±0.88a	43.58±0.93b	28.37±1.14c	58.24±2.68a	71.63±1.14a
2022-2023	MWF1	8.23±0.60b	18.43±1.51b	16.74±1.65b	41.03±2.08b	83.26±1.65b
	MWF2	6.83±0.79b	14.75±1.70c	12.45±1.43c	48.02±0.74a	87.55±1.43a

	MWF3	11.96±1.28a	22.75±1.80a	20.99±2.06a	45.00±1.41a	79.01±2.06c
	SWF1	21.21±2.74c	38.43±4.93a	42.53±4.71b	28.74±4.09b	57.47±4.71a
	SWF2	29.45±1.20a	43.88±1.87a	59.71±2.75a	19.89±1.63c	40.29±2.75b
	SWF3	25.12±1.18b	39.84±1.76a	38.62±1.58b	39.93±1.00a	61.38±1.58a
	PWF1	25.17±2.48ab	44.16±3.32ab	45.58±3.47a	30.04±2.48b	54.42±3.47b
	PWF2	27.20±0.91a	45.26±0.59a	47.19±3.23a	30.53±2.90b	52.81±3.23b
	PWF3	23.16±1.82b	39.73±2.64b	35.40±2.13b	42.27±2.43a	64.60±2.12a
Treatment		*	*	*	*	*
Year		*	*	*	*	*
Treatment*Year		*	*	*	*	*

Means in a column for the same crop followed by different lowercase letters indicate a significant difference at $p < 0.05$. * means significant difference at the 0.05 level.
